# Interferon-alpha competing endogenous RNA network antagonizes microRNA-1270

**DOI:** 10.1007/s00018-015-1875-5

**Published:** 2015-03-07

**Authors:** Tominori Kimura, Shiwen Jiang, Noriyuki Yoshida, Ryou Sakamoto, Mikio Nishizawa

**Affiliations:** 1 Department of Pharmacy, Laboratory of Microbiology and Cell Biology, College of Pharmaceutical Sciences, Ritsumeikan University, Kusatsu, Shiga 525-8577 Japan; 2 Department of Biomedical Sciences, Laboratory of Medical Chemistry, College of Life Sciences, Ritsumeikan University, Kusatsu, Shiga 525-8577 Japan

**Keywords:** Non-coding RNA, Regulatory RNA network, Competing endogenous RNA, Natural antisense RNA, Mechanisms of action

## Abstract

A new form of circuitry for gene regulation has been identified in which RNAs can crosstalk by competing for shared microRNAs (miRNAs). Such competing endogenous RNAs (ceRNAs) form a network via shared miRNA response elements (MREs) to antagonize miRNA function. We previously reported natural antisense RNA (AS) as an important modulator of interferon-α1 (IFN-α1) mRNA levels by promoting IFN-α1 mRNA stability. We show that IFN-α1 AS forms a ceRNA network with specific IFN-α AS (IFN-α7/-α8/-α10/-α14) and mRNA (IFN-α8/-α10/-α14/-α17) subtypes from the *IFN*-*α* gene (*IFNA*) family to antagonize miRNA-1270 (miR-1270), thereby modulating IFN-α1 mRNA levels. Bioinformatic analysis demonstrated that IFN-α1 AS harbors multiple miR-1270 MREs (MRE-1270s), whose presence was substantiated by miR-1270 overexpression and transfection of antimiR-1270. The antimiR-1270, complementary to the miR-1270 seed region, revealed that IFN-α1 AS likely shares the MRE-1270 with IFN-α1 mRNA and specific IFN-α AS and mRNA subtypes. Subsequent bioinformatic analysis for MRE-1270s showed that IFN-α1 AS and other RNA subtypes shared the 6-mer MRE-1270 site. Further MRE-mapping demonstrated that the total number of MRE-1270s in IFN-α1 AS accounted for approximately 30 % of the miR-1270 population. AntimiR-1270 transfection also caused specific de-repression of five cellular mRNAs, including that of *CAPRIN1*. These results suggest that IFN-α1 AS, together with specific IFN-α AS and mRNA subtypes, as well as the five cellular mRNAs, participate as competing molecules in the ceRNA network against miR-1270. This coordinated regulatory architecture suggests a vital function for the innate immune system in maintaining precise physiological type I IFN levels via post-transcriptional regulatory mechanisms.

**Electronic supplementary material:**

The online version of this article (doi:10.1007/s00018-015-1875-5) contains supplementary material, which is available to authorized users.

## Introduction

Until recently, the main role of RNA was considered to be that of an intermediary between the DNA code and its final incarnation as protein [1]. However, this hardly captures the complex interplay between the biomolecular trio of life, as has been made clear by the recent advances made by genome studies; the Encyclopedia of DNA Elements (ENCODE) project reported that three-quarters of the human genome was capable of being transcribed [2], of which merely 2.94 % was transcribed into protein-coding mRNAs [3]. This indicates that contrary to central dogma [1], non-coding RNAs (ncRNAs) represent most of the human transcriptome.

The importance of this non-coding transcriptome has become increasingly clear in recent years. Comparative genomic analysis has demonstrated a significant difference in genome utilization among species; e.g., the protein-coding genome constitutes almost the entire genome of unicellular yeast, but only 2 % of the mammalian genome [4]. Furthermore, the non-coding transcriptome is often dysregulated in cancer [5]. These observations suggest that the transcriptome is of crucial importance in the greater complexity of higher eukaryotes and in mechanisms of pathogenesis [6, 7]. Assigning function to non-coding sequence will, therefore, undoubtedly lead to important insight about basic physiology and disease progression [8].

ncRNAs comprise multiple classes of RNA transcripts that regulate transcription, stability or translation of protein-coding genes in the mammalian genome [9]. Natural antisense transcripts (NATs) form such a class and are transcribed from the opposite strand of protein-coding or non-protein-coding (sense) genes. The GENCODE v7 catalog contains 9640 long non-coding RNA (lncRNA) loci, representing 15,512 transcripts, of which 3214 are antisense loci [10].

Recently we have identified and characterized in detail one such sense–antisense pair, interferon-α1 (IFN-α1) mRNA and its antisense partner IFN-α1 AS. We demonstrated a critical role of this non-coding NAT in the post-transcriptional regulation of the IFN-α1 gene (*IFNA1*), and subsequently, IFN-α1 protein production [11]. A ~4 kb, spliced IFN-α1 AS targets a single-stranded region, called the bulged-stem loop (BSL) within a conserved secondary structure element formed in the IFN-α1 mRNA-coding region, but not in the usual 3′-untranslated region (3′-UTR) [12]. Upon recognition of the BSL region, the cytoplasmic sense–antisense interaction between the complementary transcripts results in stabilization of the IFN-α1 mRNA [11]. Here, we report evidence that IFN-α1 AS forms a competing endogenous RNA (ceRNA) network with specific IFN-α AS RNA and mRNA subtypes from the *IFNA* multigene family, as well as with several other cellular transcripts, to antagonize miRNA-1270-induced destabilization of IFN-α1 mRNA.

The mechanisms by which NATs regulate gene expression are largely unknown. Cytoplasmic sense–antisense duplex formation can alter sense mRNA stability and translation efficiency. The region of overlap between mRNAs and NATs might affect mRNA stability to reduce mRNA decay (this decay is via endo- or exonucleolytic degradation by various RNases; reviewed by Nishizawa et al. [13, 14]). Indeed, we have previously demonstrated that NATs for the gene encoding inducible nitric oxide synthase (*iNOS*), an important gene in inflammatory diseases, increase the stability of *iNOS* mRNA [15]. Enhancement of *iNOS* mRNA stability was mediated through interactions of the NAT molecules with the AU-rich element (ARE)-binding human antigen R (HuR) protein. HuR could, in turn, suppress RNA degradation by inhibiting deadenylation or exonucleases [15]. In contrast, alterations in the secondary structure of hypoxia-induced factor 1α (*HIF1A*) mRNA by an NAT could reduce the stability of the mRNA by exposing the ARE and making the RNA prone to degradation [16].

We recently demonstrated that IFN-α1 AS was involved in determining post-transcriptional *IFNA1* mRNA levels [11]. Overexpression of IFN-α1 AS in pLKO-null [17] -virus-transduced Namalwa cells consistently raised IFN-α1 mRNA levels after Sendai virus (SeV) infection. pLKO-miR-1270 virus-transduced cells had significantly reduced IFN-α1 mRNA levels, whereas subsequent overexpression of IFN-α1 AS returned the mRNA to basal levels. These results thus suggest that IFN-α1 AS and miR-1270 may compete for binding to IFN-α1 mRNA [11].

According to recent literature ([8] and references therein), this proposed miRNA masking effect would apply only to a specific set of RNA competitors, the abundance of which and of corresponding targets, should fall within a narrow, equimolar range, as the expression of competitor RNAs would have little impact on the regulation of highly abundant miRNAs. Since the relative expression levels of IFN-α1 AS were 4–6 % of IFN-α1 mRNA levels [11], we decided to investigate whether the AS itself harbors binding sites for miR-1270, which targets IFN-α1 mRNA. These binding sites are termed miR-1270 response elements or MRE-1270s [18] and act as a target RNA decoy or a ceRNA [19–21] against miR-1270.

Furthermore, the *IFNA* multigene family consists of 13 functional subtypes that show high sequence similarity to *IFNA1* (see below). Since all the IFN-α mRNA subtypes are expressed in Namalwa cells after SeV infection [22], we investigated whether IFN-α1 AS may form an antimiR-1270 ceRNA network [8] with the other IFN-α mRNA subtypes and their antisense partners. We also investigated whether other cellular mRNAs that share MRE-1270s participate in the network.

## Materials and methods

### Cell culture and virus propagation

Human Namalwa B cells (B lymphocytes from Burkitt’s lymphoma; ATCC CRL-1432) were maintained as previously described [11]. SeV was propagated as described [11] using embryonated chicken eggs. The hemagglutination titer of SeV was determined by serial titration of virus stocks in eggs.

### Plasmid construction

For the construction of plasmids for mapping MRE-1270s on the IFN-α1 mRNA and IFN-α1 AS RNA, phuIFN-α1 AS/exon 1.1, phuIFN-α1, phuIFN-α1/SL2Revertant (R), /BSLRevertant (/BSLR), and /5′-UTRRevertant (/5′-UTRR) expression plasmids [11] were digested with *Hind*III/*Xba*I and then blunt end ligated into the blunt-ended *Xba*I site of the pEF-*Luc* reporter plasmid [15], to generate pEF-*Luc*-IFN-α1-AS/exon 1.1, -mRNARevertant (-mRNAR), -SL2 and -SL2R, -BSL and BSLR, and -5′-UTRR, respectively. The orientation of the inserts was confirmed by DNA sequencing (data not shown). For the construction of pEF-*Luc*-IFN-α1-SL2/∆BSL, inverse polymerase chain reaction (PCR) was employed to generate the internal deletion mutant using the primer pair of ∆BSL-invF1 and ∆BSL-invR1. The inverse PCR-based mutagenesis was performed using the KOD-plus mutagenesis kit (TOYOBO, Osaka, Japan), according to the manufacturer’s instructions. For the construction of pEF-*Luc*-IFN-α1-AS/exon 0.1, -3′-UTRR-DSRevertant (-3′-UTRR-DSR), -(SL2-stop codon)Revertant (R), and -(ATG-SL1)Revertant (R), the human *IFNA1* AS nucleotide sequence, encoding the 0.1 kb exon, the *IFNA1*-3′-untranslated region (3′-UTR) as well as the downstream fragment (DS) of the 3′-UTR (3′-UTR-DS), (SL2-stop codon)R and (ATG-SL1)R were amplified by PCR using Namalwa cell genomic DNA as template [11]. The gene-specific primer pairs were: exon 0.1F and exon 0.1R to generate pEF-*Luc*-IFN-α1-AS/exon 0.1; 3′ UTR-F and DSR to generate pEF-*Luc*-IFN-α1-3′UTRR-DSR; SL2-stop F and SL2-stop R to generate pEF-*Luc*-IFN-α1-(SL2-stop codon)R; ATG-SL1 F and ATG-SL1 R to generate pEF-*Luc*-IFN-α1-(ATG-SL1)R. To construct pEF-*Luc*-CAPRIN1 3′-UTR, total RNA from Namalwa cells was reverse transcribed with the *CARIN1* R1 primer and cDNA was amplified by PCR using the *CAPRIN1* F and R primer pair. The gene-specific primers used are listed in Supplementary Table 1. All the amplicons were digested with *Xba*I and inserted into the *Xba*I site of pEF-*Luc*. The orientation of the inserts was confirmed by DNA sequencing (data not shown). Human chromosome 9 fragments containing *IFNA7*, *IFNA8*, *IFNA10* or *IFNA14* were amplified by PCR using the gene-specific primer pairs listed in Supplementary Table 1 and Namalwa cell genomic DNA as template. Each amplicon was then digested with *Hind*III/*Xba*I and cloned into the *Hind*III/*Xba*I sites of pSI [11] to generate phuIFN-α7, -α8, -α10 and -α14 expression vectors. For phuIFN-α17, see Kimura et al. [11].

### Oligonucleotides

Unconjugated locked nucleic acid (LNA)-modified antimiR-1270 oligonucleotides were synthesized with a complete phosphorothioate backbone (Gene-Design, Osaka, Japan). The sequence of antimiR-1270 was complementary to the seed region of the mature miR-1270 (nucleotides 2–9). The LNA mismatch control was designed according to the miR-1270 seed region sequence. These sequences and names are listed in Supplementary Table 1.

### Transfection, virus infection and isolation of total cellular RNA

Namalwa cells and the recombinant pLKO-miR-1270 lentivirus-transduced Namalwa cells [11] were subjected to magnet-assisted transfection, as described previously [11]. The transfection efficiencies were optimized when 3 µg of plasmid DNAs per 9.5 cm^2^ well was used at a 1:1 ratio with the MATra™ (IBA GmbH, Göttingen, Germany) transfection reagent for complex formation. At either 6 or 20 h after transfection, the transfected Namalwa cells were then washed and infected with 50 hemagglutination units of SeV/10^6^ cells for 60 min. The transfected and infected Namalwa cells, and the transfected pLKO-miR-1270 lentivirus-transduced Namalwa cells were further incubated at 37 °C for 24 and 48 h, respectively. Total cellular RNA was then isolated from cells, as described previously [11]. Namalwa cells were transfected as described above with both the LNA nucleotides (333 nM final concentration) and pEF-*Luc* (400 ng) and incubated for 6 h. The cells were then washed and infected with SeV. After 24 h, total cellular RNA was isolated as described above.

### Strand-specific reverse-transcription quantitative polymerase chain reaction (RT-qPCR)

RT-qPCR was performed essentially as described previously [11]. Details of the primer sequences and locations within the corresponding genes are shown in Supplementary Table 1. The results are based on cycle threshold (*C*_t_) values. Differences between the *C*_t_ values for experimental and reference (Human 18S rRNA) genes were calculated as ∆∆*C*_t_ [11]. Alternatively, using the samples shown in Fig. 3c, the copy numbers of IFN-α1 AS or mRNA were determined [11] to calculate the number of MRE-1270s. To determine miR-1270 copy number, total cellular RNA that was highly enriched for small RNAs was prepared from the samples used to produce the data in Fig. 3c using a mirVana™ miRNA isolation kit (Ambion Inc., Austin, TX, USA) according to the manufacturer’s instructions. The total cellular RNA was then reverse transcribed with specific primers using the TaqMan MicroRNA Reverse Transcription Kit (Applied Biosystems, Carlsbad, CA, USA), and real-time PCR was conducted using the TaqMan MicroRNA Assay Kit for either hsa-miR-1270 or U6 snRNA (Applied Biosystems) according to the manufacturer’s instructions. U6 snRNA was employed as an internal standard to normalize the total cellular RNA samples. Tenfold dilutions of a known concentration of a synthetic miR-1270 standard: 5′-CUGGAGUAUGGAAGAGCUGUGU-3′ (Gene-Design) were assayed in the same run using the TaqMan MicroRNA Assay Kit. The regression lines from each dilution curve were used to determine the miR-1270 copy numbers in each sample.

### Assessment of the cross-reactivity of mRNA/AS RNA PCR primer pairs for an *IFNA* subtype against the other family subtypes and of the sequence homology of *IFNA1* against other *IFNA* family genes

Sequence alignment analyses of mRNA/AS PCR primer pairs for an individual *IFNA* family subtype and for *IFNA1* were performed against the other genes in the family using a function of GENETYX-MAC software (Ver. 15.0.0) (Genetyx Corporation, Tokyo, Japan).

### Statistical analysis and informatics

Results in the figures are representative of at least three independent experiments with multiple samples (*n*: 4–6) generating similar findings. Differences presented in the figures were analyzed using Student’s *t* test.

### Accession numbers

Gene accession numbers deposited in DDBJ/EMBL/GenBank were as follows: AB578886 (human *IFNA1*), AB578885 (human *IFNA1* AS). The sequence numbering presented in this report is based on the following DDBJ/EMBL/GenBank human sequences: *IFNA2*; NM_000605.3, *IFNA4*; NM_021068.2, *IFNA5*; NM_002169.2, *IFNA6*; NM_021002.2, *IFNA7*; NM_021057.2, *IFNA8*; *NM*_002170.3, *IFNA10*; *NM*_002171.2, *IFNA13*; NM_006900.3, *IFNA14*; NM_002172.2, *IFNA16*; NM_002173.2, *IFNA17*; NM_021268.2 and *IFNA21*; NM_002175.2, cell cycle-associated protein 1 (*CAPRIN1*), transcript variant 1, mRNA; NM_005898.4, Cathepsin E (*CTSE*), transcript variant 1, mRNA; NM_001910.3, GLI family zinc finger 2 (*GLI2*), mRNA; NM_005270.4, Keratin 40 (*KRT40*), mRNA; NM_182497.3, Lysozyme (*LYZ*), mRNA; NM_000239.2, Membrane-associated ring finger (C3HC4) 5 (*MARCH5*), mRNA; NM_017824.4, OTU deubiquitinase 3 (*OTUD3*), mRNA; NM_015207.1, RAS protein activator-like 2 (*RASAL2*), transcript variant 2, mRNA; NM_170692.2 and Tandem C2 domains, nuclear (*TC2* *N*), transcript variant 1, mRNA; NM_152332. The miRNA precursor genes employed in this study were hsa-miR-1270, -miR-1287, and -miR-483 (DDBJ/EMBL/GenBank sequences: MI0006407, MI0006349 and MI0002467, respectively).

## Results

### Validation of the miR-1270 binding site in the bulged-stem loop (BSL) region within the conserved secondary structure (CSS) of IFN-α1 mRNA by luciferase constructs

**Fig. 1:**
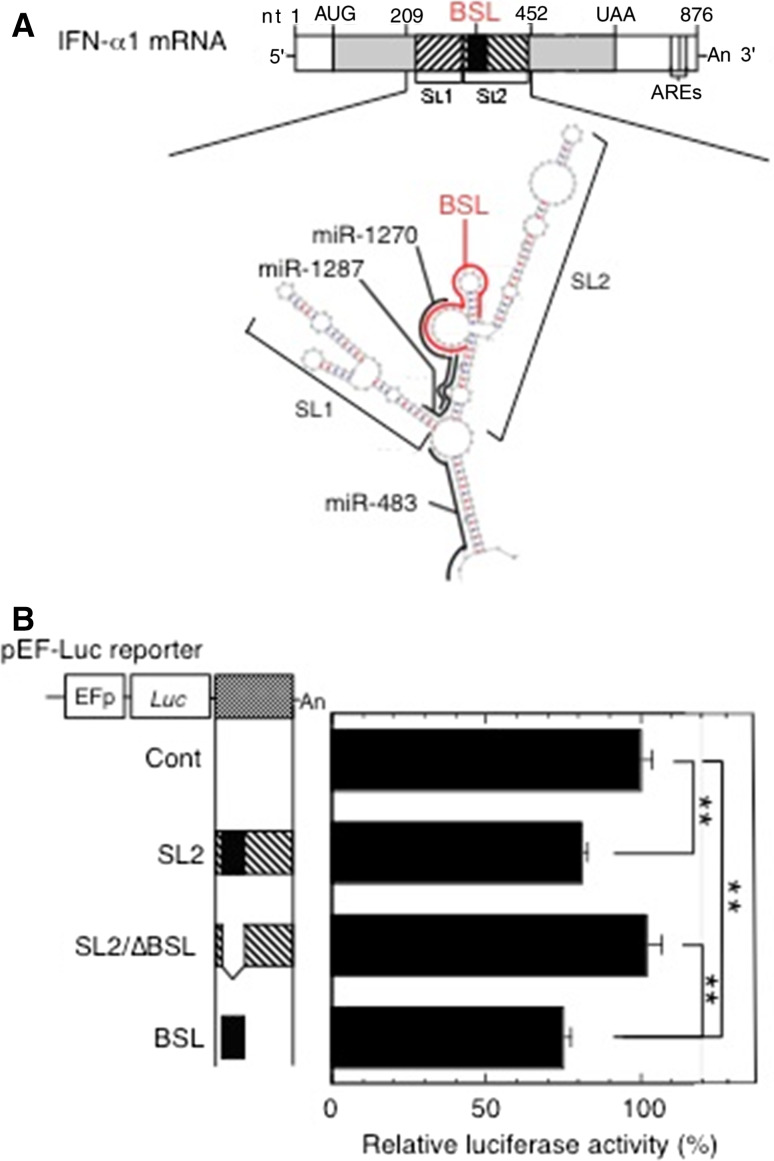
Validation of the miR-1270 response element (MRE-1270) in the BSL region of IFN-α1 mRNA. **a** The schematic shows the predicted binding sites for miR-1270 (nt 333–308), miR-1287 (nt 324–303), and miR-483 (nt 226–205) (all in *black bold lines*) and their relation to the BSL region (*IFNA1* nt 322–352; *red bold line*) in the conserved secondary structure of IFN-α1 mRNA. Figure reproduced with modification from Kimura et al. [11] with permission. **b** A series of pEF-*Luc* (luciferase) reporters harboring SL2, SL2/∆BSL or BSL of IFN-α1 mRNA were transfected into the recombinant pLKO-miR-1270 lentivirus-transduced Namalwa cells. Luciferase assays were performed 48 h after transfection as described previously [51]. The luciferase activities were normalized to the enzymatic activity determined in parental pEF-*Luc* reporter-transfected cells (Cont) and are presented as the “relative luciferase activity”, which indicates percent reporter activity relative to that of Cont as 100 %. Values of a representative experiment of three independent transfection experiments are presented as the mean ± SEM of four or five samples. **p < 0.01. The transfection efficiencies of each reporter construct shown were normalized to *Renilla* luciferase activity, an internal transfection standard

We recently reported that IFN-α1 AS prevented miRNA-induced destabilization of the IFN-α1 mRNA by masking the predicted miR-1270 binding site, thereby increasing stability of the mRNA [11]. To validate the predicted miR-1270 binding site in the IFN-α1 mRNA (Fig. 1a; reproduced from [11] with permission), we engineered the BSL-containing *IFNA1* stem loop (SL)2 fragment downstream of a luciferase reporter gene. We found that insertion of SL2 is sufficient for significant reduction of luciferase reporter activity in pLKO-miR-1270 virus-transduced cells (Fig. 1b, SL2; *p* < 0.01). Deletion of the BSL from the SL2 fragment abolished the reduction of luciferase reporter activity, whereas replacing this fragment with the BSL alone resulted in significant reduction of luciferase activity (Fig. 1b, SL2/∆BSL, and BSL; *p* < 0.01, respectively). These results indicate that the predicted miR-1270 binding site is indeed present in the BSL region, suggesting the possibility of in vivo interaction between miR-1270 and its cognate binding site, or MRE, within the CSS SL2 of the IFN-α1 mRNA.

Covering the MRE-1270 by IFN-α1 AS might eliminate miRNA-induced IFN-α1 mRNA decay. It follows that cytoplasmic sense–antisense RNA duplex formation can potentially inhibit the interactions between miR-1270 and IFN-α1 mRNA. This can explain, at least in part, the enhancement of IFN-α1 mRNA stability by IFN-α1 AS [11]. According to recent studies ([8] and references therein), this proposed miRNA masking effect would, however, only apply to a set of RNA competitors, the abundance of which and of corresponding targets fall within a narrow, equimolar range, as the expression of competitor RNAs would have little impact on regulation of highly abundant miRNAs. Furthermore, it has become clear that non-coding transcripts sharing MREs with coding transcripts can be similarly targeted, sequestering miRNAs to prevent them from acting on the protein-coding mRNAs, resulting in a complex network of ceRNAs to indirectly modulate each other’s abundance through the shared MREs ([18] and references therein).

Since the relative expression levels of IFN-α1 AS were only 4–6 % of IFN-α1 mRNA levels [11], we investigated the possibility that IFN-α1 AS might also function through its ability to act as a ceRNA in addition to, or as an alternative way of competing with miR-1270 for IFN-α1 mRNA [20]. Such activity would in turn affect the distribution of miR-1270 on its targets, thereby modulating the de-repression of miRNA targets through the MREs [19, 21], resulting, in this case, in increased IFN-α1 mRNA levels.

### Validation of miR-1270 MREs in the IFN-α1 AS

**Fig. 2:**
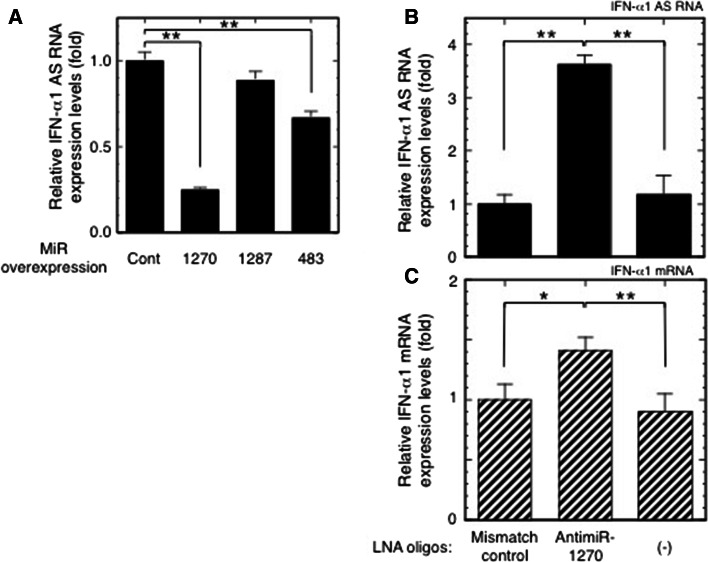
Validation of the effect of miR-1270 on IFN-α1 AS. **a** Effects of overexpressed miR-1270, miR-1287 and miR-483 on IFN-α1 AS expression were examined in SeV-infected Namalwa cells. The cells were harvested for the subsequent RT-qPCR analyses as described in the Materials and methods section. IFN-α1 AS levels were normalized to 18S rRNA and are presented as “relative IFN-α1 AS expression”, which indicates the fold change of the AS level relative to that of the control vector, [pEGFP-miR-null vector-transfected cells (Cont)] at 24 h after SeV infection. Values are presented as described in the legend to Fig. 1 (**p < 0.01). **b**, **c** Effects of inhibition of endogenous miR-1270 function on IFN-α1 AS/mRNA expression levels. LNA-modified antimiR-1270 was transfected into Namalwa cells, which were then infected with SeV and harvested for RT-qPCR analyses of relative IFN-α1 AS (**b**) and mRNA (**c**) expression as described above. The relative expressions indicate the fold change of AS/mRNA levels relative to those obtained in the LNA mismatch control-transfected, SeV-infected cells. (-) mock transfection. *p < 0.05, **p < 0.01

To validate the presence of miR-1270 MREs in the IFN-α1 AS, we overexpressed miR-1270, -1287, or -483 in SeV-infected Namalwa cells. miR-1287 and -483 target the double-stranded “stem” regions adjacent to the single-stranded BSL region (Fig. 1a) and were previously employed as negative controls in an IFN-α1 AS/miR-1270 competition assay for mRNA binding [11]. Exogenous and endogenous expression of these miRNAs was verified by quantification of each miRNA in transfected and control Namalwa cells (data not shown) (see [11] for information on primers in this assay). Similar to the effect on IFN-α1 mRNA levels [11], overexpression of miR-1270 significantly reduced IFN-α1 AS levels in the SeV-infected Namalwa cells, whereas miR-1287 had no such effect (Fig. 2a, miR-1270 O/E, *p* < 0.01; miR-1287 O/E). However, unlike IFN-α1 mRNA, overexpression of miR-483 significantly reduced IFN-α1 AS levels (Fig. 2a, miR-483 O/E, *p* < 0.01). While miR-483 reduced AS levels by 33 %, overexpression of miR-1270 caused a 75 % reduction in AS levels 24 h after viral infection. In our previous report [11], overexpression of miR-483 failed to reduce IFN-α1 mRNA levels; therefore, the results shown in Figs. 1 and 2a indicate that IFN-α1 AS and mRNA share MRE-1270.

We then assessed the effect of endogenous miR-1270 on IFN-α1 AS/mRNA expression levels by employing an antimiR approach to inhibit endogenous miR-1270 [23]. For this knockdown strategy, we designed an 8-mer antimiR-1270, a fully LNA-modified phosphorothioate oligonucleotide complementary to the seed region of miR-1270 [23]. We tested its effects on IFN-α1 AS and mRNA expression in SeV-infected Namalwa cells. Transfection of the antimiR-1270 resulted in potent antagonism of miR-1270, as shown by specific and significant de-repression of both IFN-α1 AS and mRNA expression (Fig. 2b, c) with a maximal effect observed at 333 nM (antimiR-1270 titration data not shown). Silencing endogenous miR-1270 increased IFN-α1 AS and mRNA levels by 360 and 140 %, respectively, compared to an LNA mismatch control. Overexpression of miR-1270 and inhibition of endogenous miR-1270 function by the antimiR-1270 thus indicate that compared to IFN-α1 mRNA, IFN-α1 AS is more susceptible to miR-1270-dependent degradation through the MRE, thereby potentially preventing miR-1270 from acting on IFN-α1 mRNA.

### Characterization of MRE-1270 sites in the IFN-α1 AS RNA

**Fig. 3:**
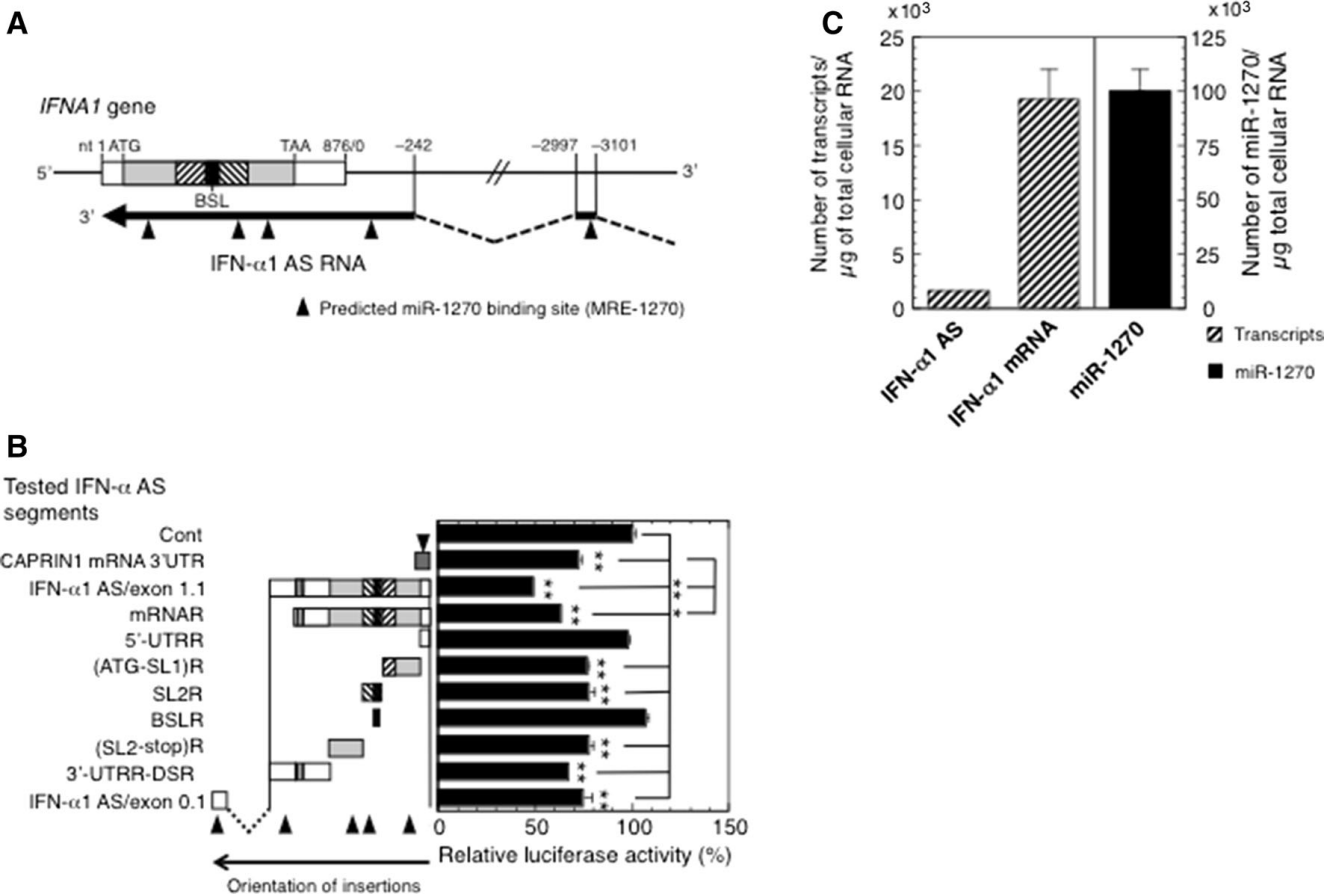
Characterization of MRE-1270s on IFN-α1 AS. **a** Location of predicted miR-1270 binding sites on IFN-α1 AS/exons 0.1 and 1.1. Bioinformatic analysis of miR-1270 binding sequences on IFN-α1 AS/exons 0.1 and 1.1 revealed the presence of five highly conserved predicted MRE-1270s (*closed triangles*). **b** Confirmation of IFN-α1 AS segments harboring the predicted MRE-1270s. A series of pEF-*Luc* reporters, into which the following IFN-α1 AS segments were inserted; IFN-α1 AS/exon 1.1, mRNA-revertant (mRNAR), 5′-UTRR, (ATG-SL1)R, SL2R, BSLR, (SL2-stop)R, 3′-UTRR-DSR, or IFN-α1 AS/exon 0.1 were transfected into pLKO-miR-1270-transduced Namalwa cells. pEF-*Luc*-CAPRIN1 3′-UTR, which harbors one MRE-1270, was included as a positive control for the assay. The luciferase activities were normalized and are presented as described in the legend to Fig. 1. The transfection efficiencies of each reporter were normalized to *Renilla* luciferase activity, an internal transfection standard. *p < 0.05, **p < 0.01. *Closed triangles* are as described in the legend to **a**. **c** Quantification of copy numbers of IFN-α1 AS/mRNA and miR-1270. Namalwa cells were infected with SeV and harvested for RT-qPCR analysis at 24 h after infection. Copy numbers of IFN-α1 AS/mRNA and miR-1270 were determined from the regression lines from the sets of standards as described in the “Materials and methods” section. The results are presented as the “Number of transcripts or miR-1270/µg of total cellular RNA” ±SEM of four or five samples. High levels of sequence homology between *IFNA1* and *IFNA13* (99.8 % in the coding region) did not allow distinction between these genes [11]. The expression levels of IFN-α1 mRNA and AS RNA presented in this study thus represent the total amounts of both IFN-α1 and -α13 mRNA/AS RNA

The relative abundance of both ceRNAs and miRNAs, their stoichiometry and the number of shared MREs are crucial determinants of ceRNA cross-regulation ([8] and references therein). We, therefore, searched for MRE-1270 motifs in the entire IFN-α1 AS RNA/exon 0.1 and 1.1 sequence. Bioinformatic analyses (RNA hybrid in BiBiserv: http://bibiserv.techfak.uni-bielefeld.de/rnahybrid/; [24], PITA: http://genie.weizmann.ac.il/pubs/mir07/index.html; [25] and MultiMiTar: http://www.isical.ac.in/~bioinfo_miu/multimitar.htm; [26]) predicted the presence of five highly conserved MRE-1270 sites in the IFN-α1 AS RNA/exons 0.1 and 1.1 (Fig. 3a and listed in Supplementary Table 4). These algorithms predict MRE-1270 sites based on sequence complementarity and free energy of the RNA duplex. We accepted free energy values that were less than −10 kcal/mol, as suggested by Zhang and Verbeek [27].

To investigate whether these motifs can actually act as MRE-1270s, the IFN-α1 AS segments that harbor these predicted MRE-1270s (Fig. 3b) were inserted downstream of the luciferase reporter gene and their effects tested on luciferase reporter activity in pLKO-miR-1270 virus-transduced cells. For this reporter assay, we included a positive control, which enabled us to quantitatively assess the relationship between the miRNA and its MREs and to analyze the effect of this relationship on the luciferase reporter gene expression.

IFN-α1 mRNA is the only target molecule for miR-1270 that has been reported [11]; therefore, we searched for positive control candidate genes that harbor potential MRE-1270s using bioinformatics analysis with the following algorithms: miRDB, http://mirdb.org/miRDB/index.html [28]; miRWalk, http://www.umm.uni-heidelberg.de/apps/zmf/mirwalk/index.html [29]; RNA22, https://cm.jefferson.edu/rna22v2.0-homo_sapiens/GetInputs.jsp [30]; Targetscan, http://www.targetscan.org/vert_61/ [31] and TargetSpy, http://www.targetspy.org/ [32]. Based on this analysis, nine genes were predicted, of which two, *CTSE* and *GLI2*, were not expressed in Namalwa cells (Supplementary Fig. 1a). Transfection of Namalwa cells with antimiR-1270 resulted in specific and significant de-repression of five out of the remaining seven mRNAs. Compared to the LNA mismatch control, silencing endogenous miR-1270 increased the expression levels of these mRNAs by between 1.2- and 1.8-fold, whereas the levels of IFN-α1 AS were increased by 4.2-fold (Supplementary Fig. 1a). Of these five mRNAs, we selected the cell cycle-associated protein 1 (*CAPRIN1*), transcript variant 1, mRNA, which showed the greatest levels of de-repression, as the positive control for the reporter assay. We inserted a segment of the *CAPRIN1* 3′-UTR, which was predicted to hold one MRE-1270, downstream of the *Luc* reporter gene and titrated the *CAPRIN1* reporter to optimize the reduction of luciferase activities in the pLKO-miR-1270-transduced Namalwa cells (Supplementary Fig. 1b). A linear correlation was observed between luciferase activity and luciferase reporter plasmid doses ranging from 0.7 to 2.2 µg; therefore, we employed 1.4 µg of each of the reporter plasmids, which caused the most significant reduction in activity, for the reporter assay shown in Fig. 3b.

The luciferase reporter assay revealed that insertion of IFN-α1 AS RNA segments that were predicted to hold one MRE-1270 each (see Supplementary Table 4), i.e., (ATG-SL1)R, SL2R, (SL2-stop)R and 3′-UTRR-DSR regions of IFN-α1 AS/exon 1.1, as well as AS/exon 0.1, reduced luciferase activity to a level comparable with that of the *CAPRIN1* positive control. In addition, insertion of both *IFNA1* AS/exon 1.1 and mRNAR caused further significant reduction in reporter signal when compared with the positive control, suggesting cumulative effects of miR-1270 on transcripts with several MREs (Fig. 3b). These results thus validate the presence of predicted MRE-1270s in the (ATG-SL1)R, SL2R, (SL2-stop)R and 3′-UTRR-DSR regions of IFN-α1 AS/exon 1.1 as well as in AS/exon 0.1.

We then measured the copy numbers of IFN-α1 AS, the mRNA and miR-1270 in SeV-infected Namalwa cells to calculate the number of MRE-1270s. In cells, the numbers of IFN-α1 AS/mRNA and miR-1270 molecules were 1558 ± 82, 19272 ± 2728 and 100100 ± 9696 per µg of total cellular RNA extracted at 24 h after viral infection, respectively (Fig. 3c). Since five IFN-α1 AS RNA segments containing five MRE-1270s were mapped to exons 0.1 and 1.1, and LNA-antimiR-1270 treatment resulted in a 3.6-fold increase of endogenous IFN-α1 AS expression levels, the number of MRE-1270s in the entire IFN-α1 AS could be no less than 1558 × 5 × 3.6 = 28044, which accounts for 28 % of miR-1270.

### Silencing miR-1270 by LNA-antimiR-1270 raised the AS/mRNA expression levels of specific subtypes of the *IFNA* multigene family

ceRNA crosstalk is optimal when the abundance of miR-1270 and ceRNA transcripts is nearly equimolar [33, 34]; therefore, we examined the effects of endogenous miR-1270 knockdown on the expression levels of mRNAs and their antisense partners from thirteen subtypes of the *IFNA* family. Strand-specific RT-PCR was employed to identify IFN-α mRNA subtypes and their AS RNAs, as successfully used previously to differentiate and identify IFN-α1 mRNAs and AS RNAs [11].

*IFNA1* has high sequence similarity with the other *IFNA* subtypes (Supplementary Table 2); therefore, prior to conducting the strand-specific RT-PCR analysis of each IFN-α AS/mRNA, a homology search of the PCR primer pairs used to detect any specific IFN-α AS and mRNA subtype was performed against other subtypes of the *IFNA* family (Supplementary Table 3). The presence of contaminating IFN-α cDNA signals was subsequently examined using plasmids that contain cDNAs, encoding genes with high, medium or low homology to the specific *IFNA* gene (Supplementary Fig. 2) [11].

**Fig. 4:**
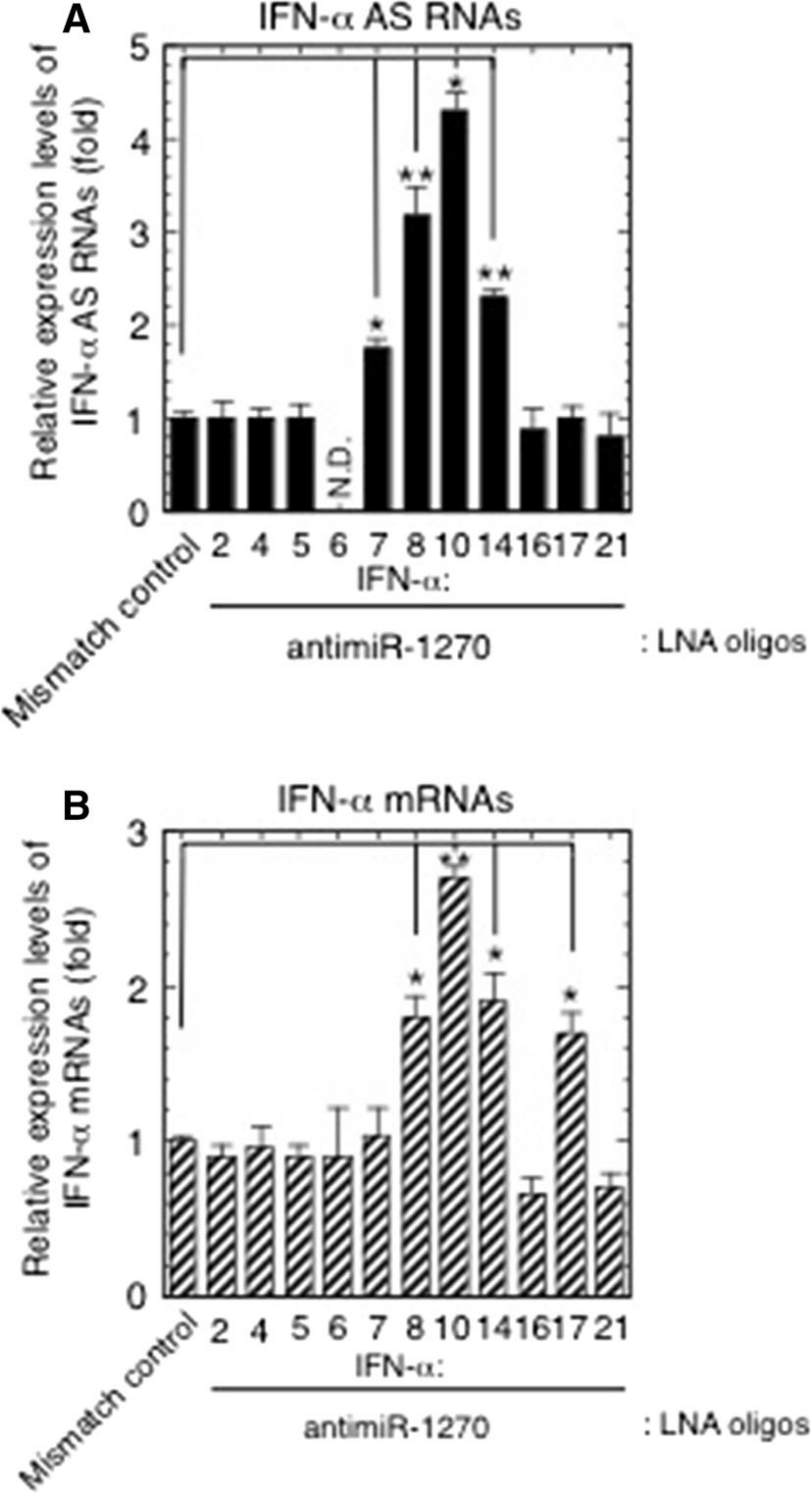
Effects of knockdown of endogenous miR-1270 function on the expression levels of IFN-α AS RNAs and mRNAs. **a**, **b** The LNA-modified antimiR-1270-transfected Namalwa cells used for the analysis in Fig. 2b, c were analyzed for the expression levels of IFN-α AS RNAs (**a**) and mRNAs (**b**) from the *IFNA* multigene family. The relative expressions indicate the fold change of AS/mRNA expression levels relative to those obtained from the LNA mismatch control-transfected cells. *p < 0.05, **p < 0.01

As previously reported [11], each plasmid was digested at either the 5′ or 3′ end and used as a template to identify contaminating antisense or sense RNA signals. The amount of any specific *IFNA* plasmid was then titrated to produce sense or antisense cDNA signals close to those obtained from IFN-α mRNA and AS of the corresponding subtype, respectively. This amount was then applied to amplifications using plasmids with high, medium or low homology. As shown in Supplementary Fig. 2, the primer pairs designed to amplify AS RNAs of IFN-α7, -α8, -α10 and -α14, as well as mRNAs of -α8, -α10, -α14 and -α17 did not cross react with any of the three antisense or sense cDNA mimics examined (the cross amplification assay was not performed against those *IFNA* family subtypes for which the antimiR-1270 treatment did not alter the RNA expression levels; Fig. 4a, b).

The strand- and *IFNA* family member-specific RT-qPCR, using the same samples used for the analysis shown in Fig. 2b, c, revealed that while transfection of the LNA oligodeoxynucleotides complementary to nucleotides 2–9 of mature miR-1270 resulted in specific de-repression of miR-1270 targets, IFN-α1 AS/mRNA (Fig. 2b, c), the miR-1270 antagonism also caused a significant increase in AS/mRNA expression levels for specific subtypes of the *IFNA* family.

As shown in Fig. 4a, b, these subtypes were IFN-α7, -α8, -α10 and -α14 AS RNAs as well as IFN-α8, -α10, -α14 and -α17 mRNAs. The increase in AS expression levels ranged from 176 % for IFN-α7 to 430 % for IFN-α10, relative to the mismatch control, whereas mRNA increases were from 170 % for IFN-α17 to 270 % for IFN-α10 relative to the mismatch control. The antimiR-1270 treatment raised the IFN-α1 AS levels by 3.6-fold (Fig. 2b), whose MRE-1270s accounted for approximately 30 % of miR-1270s. Furthermore, bioinformatic analysis using the tools employed for Fig. 3a revealed that the de-repressed IFN-α AS RNA/mRNA subtypes shared multiple MRE-1270 sites with IFN-α1 AS, of which one to three corresponding binding site sequences were identical or very similar with those of either IFN-α1 AS/exon 1.1 DSR or/exon 1.1, stop-SL2 listed in Supplementary Table 4 (see Table 1). These MRE-1270 sites have been classified as the six-nucleotide site matching the seed region [35].

**Table 1:**
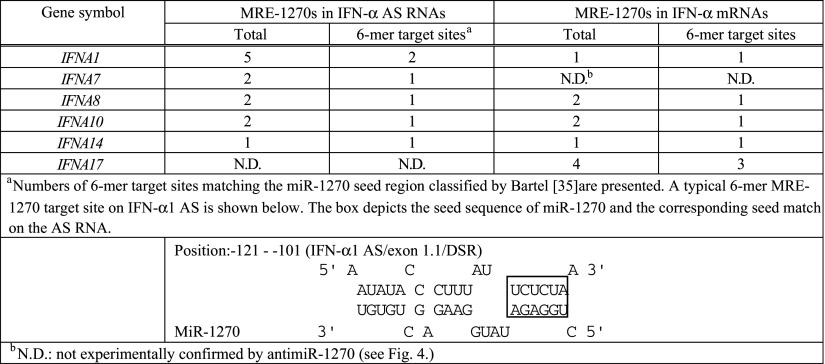
Number of predicted MRE-1270s in subtypes of the *IFNA* multigene family

The above results thus suggest that these specific subtypes of IFN-α AS RNAs/mRNAs would be able to fulfill the difference between the number of MRE-1270s in the IFN-α1 AS and the number of miR-1270 molecules as members of the interferon-α ceRNA network against the miRNA.

## Discussion

Similar to most cytokines, type I IFNs, namely IFN-α and IFN-β, induce balanced responses in which activating signals that induce antiviral states and promote immune responses are counterbalanced by suppressive signals that limit toxicity to the host and enable coexistence with chronic pathogen infection. These balanced responses are fine-tuned by host factors at multiple levels, including signaling, transcriptional, post-transcriptional and translational levels to sculpt immune responses that are appropriate for host defense and survival [36]. Aberrant or excessive stimulation of any of these processes is believed to underlie many inflammatory autoimmune disorders. For example, RNA- and DNA-associated autoantigens in systemic lupus erythematosus have been shown to drive pathological expression of type I IFN genes and IFN-induced genes through Toll-like receptor (TLR)7 and TLR9 activation [37]. The levels of type I IFNs, therefore, require tight regulation to maintain a narrow window between essential and pathological IFN expression.

We have previously demonstrated that IFN-α1 AS enhances the stability of IFN-α1 mRNA [11]. The data presented here argue for an additional, synergistic role for IFN-α1 AS in the post-transcriptional regulation of human IFN-α1 mRNA stability. We present several lines of evidence to show that IFN-α1 AS functions as a ceRNA to prevent miR-1270 from acting on IFN-α1 mRNA. (1) Overexpression of miR-1270 and transfection of antimiR-1270 revealed that IFN-α1 AS is likely to share the miR-1270 response elements (MRE-1270s) with IFN-α1 mRNA. Interactions between IFN-α1 AS and miR-1270 through the response elements would, therefore, titrate away miR-1270 from IFN-α1 mRNA, suggesting that the AS acts as an endogenous decoy or ceRNA for IFN-α1 mRNA. Indeed, antimiR-1270-dependent silencing of endogenous miR-1270 caused de-repression of IFN-α1 AS expression levels by 360 %, whereas the same treatment raised IFN-α1 mRNA levels by 40 %, indicating that the ceRNA functions as a decoy to protect the miRNA target.

It has been reported that optimal ceRNA-mediated cross-regulation occurs at a near-equimolar equilibrium between all ceRNA-MREs and miRNAs within a network [33]. However, all mammalian miRNA-competing RNAs identified so far have only one or two binding sites for the same miRNA and are not highly expressed, limiting their potency [19, 21, 38–43]. (2) We, therefore, determined the number of IFN-α1 AS segments that harbor the MRE. Contrary to previous reports, the AS/exons 0.1 and 1.1 were found to host five segments with five MREs in total. Since the copy number of IFN-α1 AS was estimated to be 1558 ± 82 per µg of total cellular RNA at 24 h after SeV infection, the overall number of MRE-1270s in AS/exons 0.1 and 1.1 would account for approximately 30 % of the miR-1270 population at the peak expression of the NAT [11]. (3) To further fill the observed gap between the number of ceRNA-MREs and the number of miR-1270 molecules, we examined the effects of antimiR-1270 on the expression levels of AS RNAs and mRNAs from other *IFNA* family subtypes, as well as on the expression levels of other cellular mRNAs that share the MRE-1270.

The antimiR-1270 sequence is complementary to the seed region of the mature miR-1270; therefore, in antimiR-1270-transfected Namalwa cells, the abundance of miR targets rises sufficiently to titrate out the miR-1270, resulting in de-repression of all the targets of this miRNA [41]. These titration effects can explain the antimiR-1270 overexpression results; specific de-repression of subtypes of both IFN-α AS RNAs (IFN-α7, -α8, -α10 and -α14 AS RNAs) as well as IFN- α mRNAs (IFN-α8, -α10, -α14 and –α17 mRNAs). The transfection of antimiR-1270 also caused specific de-repression of five other miR-1270 target mRNAs, including that of *CAPRIN1*.

Recent studies have demonstrated that miRNA competition extends beyond the non-coding transcriptome and potentially confers an additional non-protein-coding function to protein-coding mRNAs ([44]; [8] for a review); therefore, in addition to the IFN-α1 AS, these IFN-α mRNA subtypes and related antisense RNAs, as well as specific cellular mRNAs, would also participate as competing molecules in the IFN-α ceRNA network against miR-1270.

**Fig. 5:**
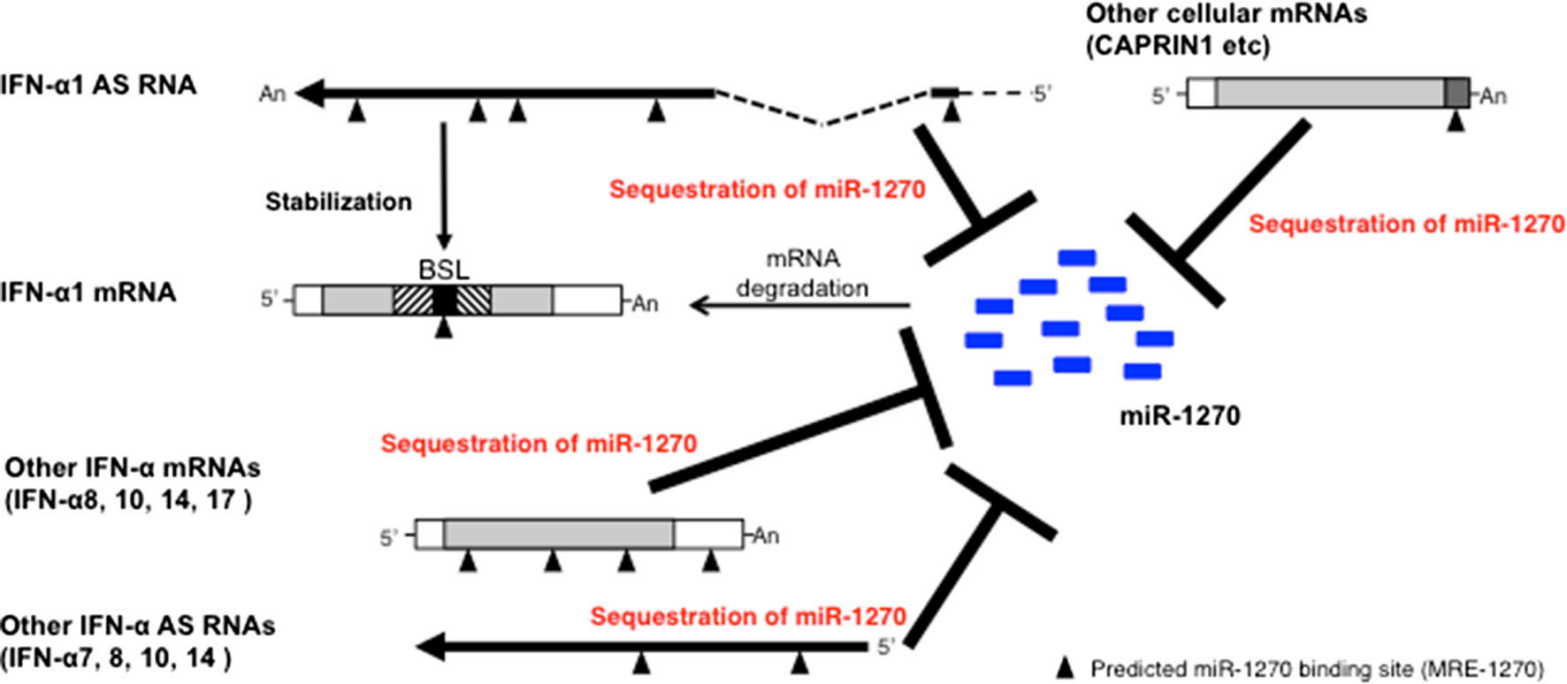
Model for an IFN-α ceRNA network against miR-1270. Following SeV infection, specific subsets of IFN-α AS RNAs (-α1, -α7, -α8, -α10 and -α14) as well as IFN-α mRNAs (-α8, -α10, -α14 and -α17), which share MRE-1270s, form a ceRNA network to titrate away miR-1270 from IFN-α1 mRNA, allowing for its expression. Several cellular mRNAs, including *CAPRIN1* mRNA, which share MRE-1270s, also participated in the network. Furthermore, upon recognition of the IFN-α1 mRNA/BSL region, IFN-α1 AS increases the stability of the mRNA [11]

The degree of specific de-repression of RNA expression levels by antimiR-1270 exceeded that of IFN-α1 AS (360 %) and mRNA (140 %) in IFN-α10 AS (430 %) as well as all the IFN-α mRNA subtypes described above (170–270 %). Furthermore, those de-repressed IFN-α AS and mRNA subtypes were predicted to harbor one to three 6-mer type, MRE-1270 sites, whose sequences were identical to or very similar to those in IFN-α1 AS. The copy numbers of IFN-α mRNA subtypes that were de-repressed exceeded the IFN-α1 mRNA copy number throughout the time course examined in the SeV-infected Namalwa cells [22]. Furthermore, silencing of endogenous miR-1270 expanded the regulatory circuitry by the addition of cellular mRNAs that share the MREs; therefore, the results suggest that the overall numbers of MRE-1270s in these molecules would be sufficient to fill the gap between the number of MREs in the IFN-α1 AS and the number of miR-1270 molecules, resulting in ceRNA network-mediated cross-regulation of IFN-α1 mRNA levels (Fig. 5).

Very recently, mathematical models have indicated that the affinity of miRNAs for their targets is another crucial parameter for the establishment of ceRNA network-mediated cross-regulation of gene expression. This especially applies to miRNAs expressed at low levels, for which effective competition can only be performed by targets that contain multiple MREs with high affinity [45] (see also Fig. 3b). Apart from the total number of shared MRE-1270s discussed above, this rule might also help to explain the apparent paradox represented by IFN-α1 AS, which is involved in a reciprocal ceRNA relationship with IFN-α1 mRNA [11] (see also Fig. 2a) despite the fact that it is expressed at 15–25 times lower levels. As was suggested by Poliseno and Pandolfi [46], this exemplifies a competitor that overcomes the limitation of being poorly expressed by means of its high affinity for the IFN-α1 mRNA-targeting miR-1270.

Five mRNAs other than the IFN-α mRNA subtypes were also shown to exert coding-independent function by acting as ceRNAs to antagonize miR-1270. One of the mRNAs, *CAPRIN1*, encodes an RNA-binding protein that plays an essential role in the proliferation of immune cells, including B lymphocytes, by the regulation of RNA metabolism and translation [47]. CAPRIN1 can modulate signaling events in the IFN response leading to translational activation [48], for example in the PI3K (phosphatidylinositol-3 kinase)/Akt and Mnk (the mitogen-activated protein kinase-integrating kinase 1) pathways that are required for IFN-stimulated gene translation [49, 50]. It is, therefore, interesting to note that the ceRNA-based competition of miRNA activity may represent an additional, previously unidentified role for *CAPRIN1* mRNA as a modulator for antiviral immunity by fine-tuning the type I IFN response.

This coordinated regulatory architecture suggests that it is vital for the host innate immune system to maintain precise type I IFN homeostasis via post-transcriptional regulatory mechanisms. The above data, along with our previous findings [11], indicate that the NAT-mRNA regulatory network exerts control over the expression of innate immunity by the proposed actions of IFN-α1 AS and other important members of the IFN-α ceRNA network, promoting target mRNA stability by transient duplex formation and inhibiting miR-1270-induced mRNA decay. These regulatory mechanisms play a pivotal role in determining the biological outcomes of type I IFN responses and whether pathogens are cleared effectively or chronic infection or autoimmune disease ensues.

### Electronic supplementary material

Below is the link to the electronic supplementary material.

Supplementary material 1 (PDF 53 kb)

Supplementary material 2 (PDF 132 kb)

Supplementary material 3 (PDF 69 kb)

Supplementary material 4 (TIFF 1402 kb)

Supplementary material 5 (TIFF 1402 kb)

Supplementary material 6 (PDF 93 kb)

## Abbreviations

miRNA: microRNA
miR-1270: miRNA-1270
ceRNA: Competing endogenous RNA
MRE: miRNA response element
AS: Antisense RNA
IFN-α1: Interferon-α1
*IFNA*: *Interferon*-*α* gene
MRE-1270: miR-1270 response element
ncRNA: Non-coding RNA
NAT: Natural antisense transcript
BSL: Bulged-stem loop
3′-untranslated region: 3′-UTR
lncRNA: Long non-coding RNA
ARE: AU-rich element
HuR: Human antigen R
*HIF1A*: Hypoxia-induced factor 1α
SeV: Sendai virus
R: Revertant
DS: Downstream fragment of *IFNA1* 3′ UTR
DSR: Antisense fragment downstream of *IFNA1* 3′ UTR
PCR: Polymerase chain reaction
LNA: Locked nucleic acid
RT-qPCR: Reverse-transcription quantitative polymerase chain reaction
Ct: Cycle threshold
CSS: Conserved secondary structure
SL: Stem loop
TLR: Toll-like receptor
*Luc*: Luciferase
*CAPRIN1*: Cell cycle-associated protein 1, transcript variant 1
*CTSE*: Cathepsin E (CTSE) transcript variant 1
*GLI2*: GLI family zinc finger 2
*KRT40*: Keratin 40
*LYZ*: Lysozyme
*MARCH5*: Membrane-associated ring finger (C3HC4) 5
*OTUD3*: OTU deubiquitinase 3
*RASAL2*: RAS protein activator-like 2, transcript variant 2
*TC2N*: Tandem C2 domains, nuclear, transcript variant 1
PI3K: Phosphatidylinositol-3 kinase
Mnk: Mitogen-activated protein kinase-integrating kinase 1
