# Supplementary material for: Interferon-alpha competing endogenous RNA network antagonizes microRNA-1270
Source: Cell Mol Life Sci. 2015 Mar 7;72(14):2749–61. doi: 10.1007/s00018-015-1875-5 (PMC4477080; doi:10.1007/s00018-015-1875-5)
Supplement: Supplementary file 1 — Supplementary material 1 (PDF 53 kb) [file 18_2015_1875_MOESM1_ESM.pdf]

Supplementary Table 2 Homology analysis of *IFNA* family genes

Gene Symbol	Accession number	CDS	Homology	Gene length, bp	Homology
IFNA1	AB578886.1	68 - 637	570 / 570 (100%)	876	876/ 876 (100%)
IFNA2	NM_000605.3	69 - 635	510 / 570 (89.5%)	1143	709/ 883 (80.3%)
IFNA4	NM_021068.2	69 - 638	503 / 570 (88.2%)	982	697/ 879 (79.3%)
IFNA5	NM_002169.2	58 - 627	512 / 570 (89.8%)	700	616/ 702 (87.7%)
IFNA6	NM_021002.2	1 - 570*	518 / 570 (90.9%)	570	518/ 570 (90.9%)
IFNA7	NM_021057.2	41 - 610	495 / 570 (86.8%)	737	611/ 743 (82.2%)
IFNA8	NM_002170.3	31 - 600	499 / 570 (87.5%)	1039	664/ 853 (77.8%)
IFNA10	NM_002171.2	47 - 616	499 / 570 (87.5%)	963	671/ 857 (78.3%)
IFNA13	NM_006900.3	67 - 639	569 / 570 (99.8%)	705	699/ 705 (99.1%)
IFNA14	NM_002172.2	45 - 614	514 / 570 (90.2%)	778	650/ 782 (83.1%)
IFNA16	NM_002173.2	7 - 576	496 / 570 (87.0%)	939	638/ 817 (78.1%)
IFNA17	NM_021268.2	50 - 619	503 / 570 (88.2%)	980	682/ 866 (78.8%)
IFNA21	NM_002175.2	49 - 618	509 / 570 (89.3%)	1024	686/ 861 (79.7%)

**IFNA6* sequence (NM_021002.2) is the protein-coding sequence only.
