# Supplementary material for: Interferon-alpha competing endogenous RNA network antagonizes microRNA-1270
Source: Cell Mol Life Sci. 2015 Mar 7;72(14):2749–61. doi: 10.1007/s00018-015-1875-5 (PMC4477080; doi:10.1007/s00018-015-1875-5)
Supplement: Supplementary file 2 — Supplementary material 2 (PDF 132 kb) [file 18_2015_1875_MOESM2_ESM.pdf]

Supplementary Table 3 Homology searches of primers used to detect the indicated IFN-α1 mRNA/AS RNA[a] against *IFNA* gene subtypes.

IFN-α2 mRNA/AS RNA

Forward PCR primer: F1B, 5'-GTGGGCTGTGATCTGCCTCAAAC-3' to detect IFN-α2 mRNA/AS RNA

Gene Symbol	Homology	Sequence (Location in the gene)
IFNA2	23 / 23 (100%)	5'-GTGGGCTGTGATCTGCCTCAAAC-3' (132-154)
IFNA4	21 / 23 (91%)	5'-CTGGGCTGTGATCTGCCTCAGAC-3' (132-154)
IFNA5	21 / 23 (91%)	5'-CTGGGCTGTGATCTGCCTCAGAC-3' (121-143)
IFNA7	21 / 23 (91%)	5'-CTGGGCTGTGATCTGCCTCAGAC-3' (104-126)
IFNA8	21 / 23 (91%)	5'-CTGGGCTGTGATCTGCCTCAGAC-3' (94-116)
IFNA16	21 / 23 (91%)	5'-CTGGGCTGTGATCTGCCTCAGAC-3' (70-92)
IFNA21	21 / 23 (91%)	5'-CTGGGCTGTGATCTGCCTCAGAC-3' (112-134)
IFNA6	20 / 23 (87%)	5'-CTGGACTGTGATCTGCCTCAGAC-3' (64-86)
IFNA10	20 / 23 (87%)	5'-CTAGGCTGTGATCTGCCTCAGAC-3' (110-132)
IFNA14	20 / 23 (87%)	5'-CTGGGCTGTAATCTGTCTCAAAC-3' (108-130)
IFNA17	20 / 23 (87%)	5'-CTAGGCTGTGATCTGCCTCAGAC-3' (113-135)
IFNA1	19 / 23 (83%)	5'-CTGGGCTGTGATCTCCCTGAGAC-3' (131-153)
IFNA13	19 / 23 (83%)	5'-CTGGGCTGTGATCTCCCTGAGAC-3' (133-155)

Reverse PCR primer: R1, 5'-AGGGATGGTTTCAGCCTTTTG-3' to detect IFN-α2 mRNA/AS RNA

Gene Symbol	Homology	Sequence (Location in the gene)
IFNA2	21 / 21 (100%)	5'-AGGGATGGTTTCAGCCTTTTG-3' (299-279)
IFNA6	18 / 21 (86%)	5'-AGAGATGGCTTCAGCCTTCTG-3' (234-214)
IFNA4	17 / 21 (81%)	5'-AGAGATGGCTTGAGCCTTCTG-3' (302-282)
IFNA5	17 / 21 (81%)	5'-AGAGATGGCTTGAGCCTTCTG-3' (291-271)
IFNA8	17 / 21 (81%)	5'-AGAGATGGCTTGAGCCTTCTG-3' (264-244)
IFNA10	17 / 21 (81%)	5'-AGAGATGGCTTGAGCCTTCTG-3' (280-260)
IFNA16	17 / 21 (81%)	5'-AGAGATGGCTTGAGCCTTCTG-3' (240-220)
IFNA21	17 / 21 (81%)	5'-AGAGATGGCTTGAGCCTTCTG-3' (282-262)
IFNA1	16 / 21 (76%)	5'-AGAGATGGCTGGAGCCTTCTG-3' (301-281)
IFNA13	16 / 21 (76%)	5'-AGAGATGGCTGGAGCCTTCTG-3' (303-283)
IFNA17	12 /21 (57%)	5'-TCAAATGTTTTCAGCAGTGTA-3' (246-226)
IFNA7	11 / 21 (52%)	5'-TGGGCTGTGATCTGCCTCAGA-3' (633-613)
IFNA14	11 / 21 (52%)	5'-TCAAATGTTTTCAGGAGTGTA-3' (467-447)

IFN-α4 mRNA/AS RNA

Forward PCR primer: F1B, 5´-ACATGATTTCGGATTCCCCGAGGAGGAG-3' to detect IFN-α4 mRNA/AS RNA

Gene Symbol	Homology	Sequence (Location in the gene)
IFNA4	28 / 28 (100%)	5'-ACATGATTTCGGATTCCCCGAGGAGGAG-3' (236-263)
IFNA7	25 / 28 (89%)	5'-ACATGAATTCAGATTCCCAGAGGAGGAG-3' (208-235)
IFNA10	25 / 28 (89%)	5'-ACATGATTTCCGAATCCCCCAGGAGGAG-3' (214-241)
IFNA16	25 / 28 (89%)	5'-ATATGATTTCGGATTCCCCCAGGAGGTG-3' (174-201)
IFNA21	25 / 28 (89%)	5'-ACATGACTTTGGATTCCCCCAGGAGGAG-3' (216-243)
IFNA1	24 / 28 (86%)	5'-ACATGACTTTGGATTTCCCCAGGAGGAG-3' (235-262)
IFNA2	24 / 28 (86%)	5'-ACATGACTTTGGATTTCCCCAGGAGGAG-3' (236-263)
IFNA6	24 / 28 (86%)	5'-ACATGACTTCAGATTTCCCCAGGAGGAG-3' (168-195)
IFNA8	24 / 28 (86%)	5'-ACATGACTTTGAATTCCCCCAGGAGGAG-3' (198-225)
IFNA13	24 / 28 (86%)	5'-ACATGACTTTGGATTTCCCCAGGAGGAG-3' (237-264)
IFNA5	23 / 28 (82%)	5'-ACATGACTTTGGATTTCCTCAGGAGGAG-3' (225-252)
IFNA17	23 / 28 (82%)	5'-ACATGACTTTGGACTTCCCCAGGAGGAG-3' (217-244)
IFNA14	22 / 28 (79%)	5'-ACATGACTTTGAATTTCCCCAGGAGGAA-3' (212-239)

Reverse PCR primer: R1, 5'-TTTTTCTAGGAGGCTCTGTTCCCAAGCAGC-3' to detect IFN-α4 mRNA/AS RNA

Gene Symbol	Homology	Sequence (Location in the gene)
IFNA4	30 / 30 (100%)	5'-TTTTTCTAGGAGGCTCTGTTCCCAAGCAGC-3' (389-360)
IFNA7	30 / 30 (100%)	5'-TTTTTCTAGGAGGCTCTGTTCCCAAGCAGC-3' (361-332)
IFNA10	30 / 30 (100%)	5'-TTTTTCTAGGAGGCTCTGTTCCCAAGCAGC-3' (367-338)
IFNA17	30 / 30 (100%)	5'-TTTTTCTAGGAGGCTCTGTTCCCAAGCAGC-3' (370-341)
IFNA21	29 / 30 (97%)	5'-TTTTTCTAGGAGGCTCTGTTCCCAAGTAGC-3' (369-340)
IFNA14	27 / 30 (90%)	5'-TTTTTCTAGGAGGGTCTCATCCCAAGCAGC-3' (365-336)
IFNA2	26 / 30 (87%)	5'-TTTGTCTAGGAGGGTCTCATCCCAAGCAGC-3' (386-357)
IFNA16	26 / 30 (87%)	5'-TTTGTCTAGGAGGGTCTCATCCCAAGCAGC-3' (327-298)
IFNA1	25 / 30 (83%)	5'-TTTGTCTAGGAGGTCCTCATCCCAAGCAGC-3' (388-359)
IFNA13	25 / 30 (83%)	5'-TTTGTCTAGGAGGTCCTCATCCCAAGCAGC-3' (390-361)
IFNA6	24 / 30 (80%)	5'-TTTGTCTAGAAGCCTCTCATCCCAAGCAAC-3' (321-292)
IFNA5	23 / 30 (77%)	5'-TTTGTCTAGAAGTGTCTCATCCCAAGTAGC-3' (378-349)
IFNA8	23 / 30 (77%)	5'-TTCATCTAGAAGGGTCTCATCCAAAGCAGC-3' (351-322)

IFN-α5 mRNA/AS RNA

Forward PCR primer: F1B, 5'-CAGACCCACAGCCTGAGTAAC-3' to detect IFN-α5 mRNA/AS RNA

Gene Symbol	Homology	Sequence (Location in the gene)
IFNA5	21 / 21 (100%)	5'-CAGACCCACAGCCTGAGTAAC-3' (139-159)
IFNA4	19 / 21 (90%)	5'-CAGACCCACAGCCTGGGTAAT-3' (150-170)
IFNA6	19 / 21 (90%)	5'-CAGACCCACAGCCTGGGTCAC-3' (82-102)
IFNA7	19 / 21 (90%)	5'-CAGACCCACAGCCTGCGTAAT-3' (122-142)
IFNA8	19 / 21 (90%)	5'-CAGACTCACAGCCTGGGTAAC-3' (112-132)
IFNA10	19 / 21 (90%)	5'-CAGACCCACAGCCTGGGTAAT-3' (128-148)
IFNA14	19 / 21 (90%)	5'-CAAACCCACAGCCTGAATAAC-3' (126-146)
IFNA17	19 / 21 (90%)	5'-CAGACCCACAGCCTGGGTAAT-3' (131-151)
IFNA21	19 / 21 (90%)	5'-CAGACCCACAGCCTGGGTAAT-3' (130-150)
IFNA1	18 / 21 (86%)	5'-GAGACCCACAGCCTGGATAAC-3' (136-156)
IFNA2	18 / 21 (86%)	5'-CAAACCCACAGCCTGGGTAGC-3' (150-170)
IFNA13	18 / 21 (86%)	5'-GAGACCCACAGCCTGGATAAC-3' (151-171)
IFNA16	18 / 21 (86%)	5'-CAGACTCACAGCCTGGGTAAT-3' (88-108)

Reverse PCR primer: R1, 5'-GAATTTGTCTAGAAGTGTCTCATCCCAAGT-3' to detect IFN-α5 mRNA/AS RNA

Gene Symbol	Homology	Sequence (Location in the gene)
IFNA5	30 / 30 (100%)	5'-GAATTTGTCTAGAAGTGTCTCATCCCAAGT-3' (381-352)
IFNA2	27 / 30 (90%)	5'-GAATTTGTCTAGGAGGGTCTCATCCCAAGC-3' (389-360)
IFNA16	27 / 30 (90%)	5'-GAATTTGTCTAGGAGGGTCTCATCCCAAGC-3' (330-301)
IFNA6	26 / 30 (87%)	5'-GAGTTTGTCTAGAAGCCTCTCATCCCAAGC-3' (324-295)
IFNA14	26 / 30 (87%)	5'-GAATTTTTCTAGGAGGGTCTCATCCCAAGC-3' (368-339)
IFNA1	25 / 30 (83%)	5'-GAATTTGTCTAGGAGGTCCTCATCCCAAGC-3' (391-362)
IFNA8	25 / 30 (83%)	5'-GAATTCATCTAGAAGGGTCTCATCCAAAGC-3' (354-325)
IFNA13	25 / 30 (83%)	5'-GAATTTGTCTAGGAGGTCCTCATCCCAAGC-3' (393-364)
IFNA21	23 / 30 (77%)	5'-AAATTTTTCTAGGAGGCTCTGTTCCCAAGT-3' (372-343)
IFNA4	22 / 30 (73%)	5'-AAATTTTTCTAGGAGGCTCTGTTCCCAAGC-3' (392-363)
IFNA7	22 / 30 (73%)	5'-AAATTTTTCTAGGAGGCTCTGTTCCCAAGC-3' (364-335)
IFNA10	22 / 30 (73%)	5'-AAATTTTTCTAGGAGGCTCTGTTCCCAAGC-3' (370-341)
IFNA17	22 / 30 (73%)	5'-AAATTTTTCTAGGAGGCTCTGTTCCCAAGC-3' (373-344)

IFN-α6 mRNA/AS RNA

Forward PCR primer: F1B, 5'-CTGGGTCACAGGAGGACCATGAT-3' to detect IFN-α6 mRNA/AS RNA

Gene Symbol	Homology	Sequence (Location in the gene)
IFNA6	23 / 23 (100%)	5'-CTGGGTCACAGGAGGACCATGAT-3' (94-116)
IFNA1	20 / 23 (87%)	5'-CTGGATAACAGGAGGACCTTGAT-3' (161-183)
IFNA2	20 / 23 (87%)	5'-CTGGGTAGCAGGAGGACCTTGAT-3' (162-184)
IFNA8	20 / 23 (87%)	5'-CTGGGTAACAGGAGGGCCTTGAT-3' (124-146)
IFNA13	20 / 23 (87%)	5'-CTGGATAACAGGAGGACCTTGAT-3' (163-185)
IFNA4	19 / 23 (83%)	5'-CTGGGTAATAGGAGGGCCTTGAT-3' (162-184)
IFNA5	19 / 23 (83%)	5'-CTGAGTAACAGGAGGACTTTGAT-3' (151-173)
IFNA10	19 / 23 (83%)	5'-CTGGGTAATAGGAGGGCCTTGAT-3' (140-162)
IFNA16	19 / 23 (83%)	5'-CTGGGTAATAGGAGGGCCTTGAT-3' (100-122)
IFNA17	19 / 23 (83%)	5'-CTGGGTAATAGGAGGGCCTTGAT-3' (143-165)
IFNA21	19 / 23 (83%)	5'-CTGGGTAATAGGAGGGCCTTGAT-3' (142-164)
IFNA7	18 / 23 (78%)	5'-CTGCGTAATAGGAGGGCCTTGAT-3' (134-156)
IFNA14	18 / 23 (78%)	5'-CTGAATAACAGGAGGACTTTGAT-3' (138-160)

Reverse PCR primer: R1, 5'-CTGAATCACCTCATGGAGGACAGAG-3' to detect IFN-α6 mRNA/AS RNA

Gene Symbol	Homology	Sequence (Location in the gene)
IFNA6	25 / 25 (100%)	5'-CTGAATCACCTCATGGAGGACAGAG-3' (255-231)
IFNA1	23 / 25 (92%)	5'-CTGGATCAGCTCATGGAGGACAGAG-3' (322-298)
IFNA4	23 / 25 (92%)	5'-CTGGATCATCTCATGGAGGACAGAG-3' (323-299)
IFNA5	23 / 25 (92%)	5'-CTGGATCATCTCATGGAGGACAGAG-3' (312-288)
IFNA7	23 / 25 (92%)	5'-CTGGATCATCTCATGGAGGACAGAG-3' (295-271)
IFNA8	23 / 25 (92%)	5'-CTGGATCATCTCATGGAGGACAGAG-3' (285-261)
IFNA10	23 / 25 (92%)	5'-CTGGATCATCTCATGGAGGACAGAG-3' (301-277)
IFNA13	23 / 25 (92%)	5'-CTGGATCAGCTCATGGAGGACAGAG-3' (324-300)
IFNA14	23 / 25 (92%)	5'-CTGCATCATCTCATGGAGGACAGAG-3' (299-275)
IFNA17	23 / 25 (92%)	5'-CTGGATCATCTCATGGAGGACAGAG-3' (304-280)
IFNA21	23 / 25 (92%)	5'-CTGGATCATCTCATGGAGGACAGAG-3' (303-279)
IFNA2	22 / 25 (88%)	5'-CTGGATCATCTCATGGAGGACAGGG-3' (320-296)
IFNA16	21 / 25 (84%)	5'-CTGGATCATCTCATGGAAGGCAGAG-3' (261-237)

IFN-α7 mRNA/AS RNA

Forward PCR primer: F2, 5'-GTACTCAGCTACAAATCCATCTGCTCTC-3' to detect IFN-α7 mRNA/AS RNA

Gene symbol	Homology	Sequence (Location in the gene)
IFNA7	28 / 28 (100%)	5'-GTACTCAGCTACAAATCCATCTGCTCTC-3' (77-104)
IFNA4	26 / 28 (93%)	5'-GTGCTCAGCTACAAATCCATCTGTTCTC-3' (105-132)
IFNA10	26 / 28 (93%)	5'-GTGCTCAGCTACAAATCCATCTGTTCTC-3' (83-110)
IFNA16	26 / 28 (93%)	5'-GTGCTCAGCTACAAATCCATCTGTTCTC-3' (43-70)
IFNA17	26 / 28 (93%)	5'-GTGCTCAGCTACAAATCCATCTGTTCTC-3' (86-113)
IFNA21	26 / 28 (93%)	5'-GTGCTCAGCTACAAATCCATCTGTTCTC-3' (85-112)
IFNA1	23 / 28 (82%)	5'-GTGCTCAGCTGCAAGTCAAGCTGCTCTC-3' (104-131)
IFNA6	23 / 28 (82%)	5'-GTGCTCAGCTGCAAGTCAAGCTGCTCTC-3' (37-64)
IFNA8	23 / 28 (82%)	5'-GTGCTCAGCTACAAGTCATTCAGCTCTC-3′ (67-94)
IFNA 13	23 / 28 (82%)	5'-GTGCTCAGCTGCAAGTCAAGCTGCTCTC-3' (106-133)
IFNA14	23 / 28 (82%)	5'-GTGCTCAGCTGCAAGTCAAGCTGCTCTC-3' (81-108)
IFNA2	22 / 28 (79%)	5'-GTGCTCAGCTGCAAGTCAAGCTGCTCTG-3' (105-132)
IFNA5	22 / 28 (79%)	5'-GTGCTCAACTGCAAGTCAATCTGTTCTC-3' (94-121)

Reverse PCR primer: R2, 5'-GCCATCAAACTCCTCCTCTGGGAATCT-3' to detect IFN-α7 mRNA/AS RNA

Gene symbol	Homology	Sequence (Location in the gene)
IFNA7	27 / 27 (100%)	5'-GCCATCAAACTCCTCCTCTGGGAATCT-3' (244-218)
IFNA4	25 / 27 (93%)	5'-GCCATCAAACTCCTCCTCGGGGAATCC-3' (272-246)
IFNA6	24 / 27 (89%)	5'-GCCATCAAACTCCTCCTGGGGAAATCT-3' (204-178)
IFNA21	24 / 27 (89%)	5'-GCCATCAAACTCCTCCTGGGGGAATCC-3' (252-226)
IFNA1	23 / 27 (85%)	5'-GCCATCAAACTCCTCCTGGGGAAATCC-3' (271-245)
IFNA5	23 / 27 (85%)	5'-GCCATCAAACTCCTCCTGAGGAAATCC-3' (261-235)
IFNA10	23 / 27 (85%)	5'-GCCATCAAACTCCTCCTGGGGGATTCG-3' (250-224)
IFNA13	23 / 27 (85%)	5'-GCCATCAAACTCCTCCTGGGGAAATCC-3' (273-247)
IFNA16	23 / 27 (85%)	5'-GCCATCAAACACCTCCTGGGGGAATCC-3' (210-184)
IFNA17	22 / 27 (81%)	5'-GCCATCAAACTCCTCCTGGGGAAGTCC-3' (253-227)
IFNA8	21 / 27 (78%)	5'-ATCATCAAACTCCTCCTGGGGGAATTC-3' (234-208)
IFNA14	21 / 27 (78%)	5'-GCCATCAAATTCCTCCTGGGGAAATTC-3' (248-222)
IFNA2	19 / 27 (70%)	5'-GTTGCCAAACTCCTCCTGGGGAAATCC-3' (272-246)

IFN-α8 mRNA/AS RNA

Forward PCR primer: F1B, 5'-CTATCT-ATAGGGCTTAAATTAGTTTTGTTC-3' to detect IFN-α8 mRNA/AS RNA

Gene symbol	Homology	Sequence (Location in the gene)
IFNA8	30 / 30 (100%)	5'-CTATCT-ATAGGGCTTAAATTAGTTTTGTTC-3' (841-870)
IFNA1	24 / 30 (80%)	5'-CTATTC-ATAAGATTTAAATTATTTTTGTTC-3' (782-811)
IFNA2	22 / 28 (79%)	5'-CTATTT-ATAAAAC--AACTTATTTTTGTTC-3' (875-902)
IFNA21	22 / 30 (73%)	5'-TTATTT-ATTAGATTTAAATTATTTTTGTCC-3' (849-878)
IFNA16	20 / 30 (67%)	5'-CTATTT-TTAAGGTTTAAATCATGTTTTATG-3' (810-839)
IFNA17	20 / 30 (67%)	5'-TTATTT-TTAAGATTTAAATTATTTTTTTAT-3' (849-878)
IFNA4	19 / 30 (63%)	5'-TTATTT-TTAAAATTTAAATTATTTTTTATG-3' (864-893)
IFNA10	16 / 30 (53%)	5'-GTTTAA-ATATTTATTTAATTATTTTTAAAA-3' (827-856)
IFNA7	14 / 30 (47%)	5'-TTATCT-AATGGAGAAGAAATACAGCCCTTG-3' (496-525)
IFNA5	14 / 31 (45%)	5'-CTATCTGACAGAGAAGAAATACAGCCCTTGT-3' (513-543)
IFNA13	14 / 31 (45%)	5'-CTATCTGACAGAGAAGAAATACAGCCCTTGT-3' (525-555)
IFNA6	12 / 30 (40%)	5'-CTCTAT-ACTGAACTTTACCAGCAGCTGAAT-3' (322-351)
IFNA14		No sequence in the sense strand matched to F1B.

Reverse PCR primer:R1, 5'-CATTCTTTAATCA-GT-TTGCACAATC-3' to detect IFN-α8 mRNA/AS RNA

Gene symbol	Homology	Sequence (Location in the gene)
IFNA8	25 / 25 (100%)	5'-CATTCTTTAATCA-GT-TTGCACAATC-3' (1033-1009)
IFNA2	21 / 25 (84%)	5'-TATTCTGTAATCA-GG-TTGCACAATT-3' (1073-1049)
IFNA7	16 / 26 (62%)	5'-AATCCTTTTTTCAAGT-TTGTTGAAAA-3' (594-569)
IFNA5	14 / 25 (56%)	5'-CGATGTTGAACCA-GT-TTTCATTCCT-3' (644-620)
IFNA10	14 / 25 (56%)	5'-CCATGTTGAACCA-GT-TTTCAATCCT-3' (633-609)
IFNA16	14 / 25 (56%)	5'-CCATGTTGAATGA-GT-TTTCAATCCT-3' (593-569)
IFNA17	14 / 25 (56%)	5'-CCATGTTGAACCA-GT-TTTCAATCCT-3' (636-612)
IFNA21	14 / 25 (56%)	5'-AATAATATAAGAA-GT-TTTAATAGTA-3' (992-968)
IFNA13	14 / 26 (54%)	5'-TCCTCCTTAATCT-TTCTTGCAAGTTT-3' (631-606)
IFNA4	13 / 25 (52%)	5'-TCCTCCTTAATCT-TT-TTTGCAAGTT-3' (630-606)
IFNA6	13 / 25 (52%)	5'-CATCAGTAAAGCA-AA-AGGCAAAGCC-3' (3-27)
IFNA14	13 / 25 (52%)	5'-CACTCCTGAAAAC-AT-TTGAAAATTT-3' (751-727)
IFNA1	12 / 25 (48%)	5'-AATAGATAAATCA-GT-TTATCAGCAT-3' (766-742)

IFN-α10 mRNA/AS RNA

Forward PCR primer: F1B, 5'-ATTTCCGAATCCCCCAGGAGGAGTT-3' to detect IFN-α10 mRNA/AS RNA

Gene symbol	Homology	Sequence (Location in the gene)
IFNA10	25 / 25 (100%)	5'-ATTTCCGAATCCCCCAGGAGGAGTT-3' (219-243)
IFNA4	22 / 25 (88%)	5'-ATTTCGGATTCCCCGAGGAGGAGTT-3' (241-265)
IFNA16	22 / 25 (88%)	5'-ATTTCGGATTCCCCCAGGAGGTGTT-3' (179-203)
IFNA6	21 / 25 (84%)	5'-ACTTCAGATTTCCCCAGGAGGAGTT-3' (173-197)
IFNA21	21 / 25 (84%)	5'-ACTTTGGATTCCCCCAGGAGGAGTT-3' (221-245)
IFNA1	20 / 25 (80%)	5'-ACTTTGGATTTCCCCAGGAGGAGTT-3' (240-264)
IFNA2	20 / 25 (80%)	5'-ACTTTGGATTTCCCCAGGAGGAGTT-3' (241-265)
IFNA7	20 / 25 (80%)	5'-AATTCAGATTCCCAGAGGAGGAGTT-3' (213-237)
IFNA8	20 / 25 (80%)	5'-ACTTTGAATTCCCCCAGGAGGAGTT-3' (203-227)
IFNA13	20 / 25 (80%)	5'-ACTTTGGATTTCCCCAGGAGGAGTT-3' (242-266)
IFNA17	20 / 25 (80%)	5'-ACTTTGGACTTCCCCAGGAGGAGTT-3' (222-246)
IFNA5	19 / 25 (76%)	5'-ACTTTGGATTTCCTCAGGAGGAGTT-3' (230-254)
IFNA14	18 / 25 (72%)	5'-ACTTTGAATTTCCCCAGGAGGAATT-3' (217-241)

Reverse PCR primer: R1, 5'-TTCTAGGAGGCTCTGTTCCCAAGCA-3' to detect IFN-α10 mRNA/AS RNA

Gene symbol	Homology	Sequence (Location in the gene)
IFNA10	25 / 25 (100%)	5'-TTCTAGGAGGCTCTGTTCCCAAGCA-3' (364-340)
IFNA17	25 / 25 (100%)	5'-TTCTAGGAGGCTCTGTTCCCAAGCA-3' (367-343)
IFNA4	25 / 25 (100%)	5'-TTCTAGGAGGCTCTGTTCCCAAGCA-3' (386-362)
IFNA7	25 / 25 (100%)	5'-TTCTAGGAGGCTCTGTTCCCAAGCA-3' (358-334)
IFNA21	24 / 25 (96%)	5'-TTCTAGGAGGCTCTGTTCCCAAGTA-3' (366-342)
IFNA14	22 / 25 (88%)	5'-TTCTAGGAGGGTCTCATCCCAAGCA-3' (362-338)
IFNA16	21 / 25 (84%)	5'-GTCTAGGAGGGTCTCATCCCAAGCA-3' (324-300)
IFNA2	21 / 25 (84%)	5'-GTCTAGGAGGGTCTCATCCCAAGCA-3' (383-359)
IFNA1	20 / 25 (80%)	5'-GTCTAGGAGGTCCTCATCCCAAGCA-3' (385-361)
IFNA13	20 / 25 (80%)	5'-GTCTAGGAGGTCCTCATCCCAAGCA-3' (387-363)
IFNA6	20 / 25 (80%)	5'-GTCTAGAAGCCTCTCATCCCAAGCA-3' (318-294)
IFNA5	18 / 25 (72%)	5'-GTCTAGAAGTGTCTCATCCCAAGTA-3' (375-351)
IFNA8	13 / 25 (52%)	5'-TTCTGCTCTGACAACCTCCCAGGCA-3' (540-516)

IFN-α14 mRNA/AS RNA

Forward PCR primer: F1B, 5'-CTGGGCTGTAATCTGTCTCAA-3' to detect IFN-α14 mRNA/AS RNA

Gene Symbol	Homeology	Sequence(Location in the gene)
IFNA14	21 / 21 (100%)	5'-CTGGGCTGTAATCTGTCTCAA-3' (108-128)
IFNA2	18 / 21 (86%)	5'-GTGGGCTGTGATCTGCCTCAA-3' (132-152)
IFNA4	18 / 21 (86%)	5'-CTGGGCTGTGATCTGCCTCAG-3' (132-152)
IFNA5	18 / 21 (86%)	5'-CTGGGCTGTGATCTGCCTCAG-3' (121-141)
IFNA7	18 / 21 (86%)	5'-CTGGGCTGTGATCTGCCTCAG-3' (104-124)
IFNA8	18 / 21 (86%)	5'-CTGGGCTGTGATCTGCCTCAG-3' (94-114)
IFNA16	18 / 21 (86%)	5'-CTGGGCTGTGATCTGCCTCAG-3' (70-90)
IFNA21	18 / 21 (86%)	5'-CTGGGCTGTGATCTGCCTCAG-3' (112-132)
IFNA10	17 / 21 (81%)	5'-CTAGGCTGTGATCTGCCTCAG-3' (110-130)
IFNA17	17 / 21 (81%)	5'-CTAGGCTGTGATCTGCCTCAG-3' (113-133)
IFNA1	16 / 21 (76%)	5'-CTGGGCTGTGATCTCCCTGAG-3' (118-138)
IFNA13	16 / 21 (76%)	5'-CTGGGCTGTGATCTCCCTGAG-3' (133-153)
IFNA6	12/ 20 60%)	5'-GAAGGCTG-AAGCCATCTCTG-3' (216-235)

Reverse PCR primer: R1, 5'-AGAGATGGCTTGAGCTTTCTG-3' to detect IFN-α14 mRNA/AS RNA

Gene Symbol	Homeology	Sequence(Location in the gene)
IFNA14	21 / 21 (100%)	5'-AGAGATGGCTTGAGCTTTCTG-3' (278-258)
IFNA4	20 / 21 (95%)	5'-AGAGATGGCTTGAGCCTTCTG-3' (302-282)
IFNA5	20 / 21 (95%)	5'-AGAGATGGCTTGAGCCTTCTG-3' (291-271)
IFNA8	20 / 21 (95%)	5'-AGAGATGGCTTGAGCCTTCTG-3' (264-244)
IFNA10	20 / 21 (95%)	5'-AGAGATGGCTTGAGCCTTCTG-3' (280-260)
IFNA16	20 / 21 (95%)	5'-AGAGATGGCTTGAGCCTTCTG-3' (240-220)
IFNA21	20 / 21 (95%)	5'-AGAGATGGCTTGAGCCTTCTG-3' (282-262)
IFNA1	19 / 21 (90%)	5'-AGAGATGGCTGGAGCCTTCTG-3' (301-281)
IFNA6	19 / 21 (90%)	5'-AGAGATGGCTTCAGCCTTCTG-3' (234-214)
IFNA7	19 / 21 (90%)	5'-AGAGATGGCTTGAGTCTTCTG-3' (274-254)
IFNA13	19 / 21 (90%)	5'-AGAGATGGCTGGAGCCTTCTG-3' (303-283)
IFNA17	19 / 21 (90%)	5'-AGAGATGGCTTGAGTCTTCTG-3' (283-263)
IFNA2	16 / 21 (76%)	5'-AGGGATGGTTTCAGCCTTTTG-3' (630-610)

IFN-α16 mRNA/AS RNA

Forward PCR primer: F1B, 5'-TTTTCAGGAGTGTAAAGAAGCA-3' to detect IFN-α16 mRNA/AS RNA

Gene symbol	Homology	Sequence (Location in the gene)
IFNA16	22 / 22 (100%)	5'-TTTTCAGGAGTGTAAAGAAGCA-3' (699-720)
IFNA14	22 / 22 (100%)	5'-TTTTCAGGAGTGTAAAGAAGCA-3' (740-761)
IFNA17	20 / 22 (91%)	5'-TTTTCAGCAGTGTAAAGAAGCG-3' (742-763)
IFNA21	20 / 22 (91%)	5'-TTTTCAGGAGTGTAAGGAAACA-3' (746-767)
IFNA10	19 / 22 (86%)	5'-TTTTCAGCAGTGTAAAGAAGTG-3' (739-760)
IFNA8	19 / 22 (86%)	5'-TTTTCAGGAGTGTTAAGCAACA-3' (730-751)
IFNA4	19 / 22 (86%)	5'-TTTTCAGCAGTGTGAAGAAGCT-3' (761-782)
IFNA2	16 / 22 (73%)	5'-TTTTTAGGAGTATTAATCAACA-3' (759-780)
IFNA1	14 / 21 (67%)	5'-ATCTCAGCA-AGCCCAGAAGCA-3' (32-52)
IFNA13	14 / 21 (67%)	5'-ATCTCAGCA-AGCCCAGAAGCA-3' (34-54)
IFNA5	11 / 22 (50%)	5'-GTTCCAGAAGGCTCAAGCCATC-3' (267-288)
IFNA6	11 / 22 (50%)	5'-GTTCCAGAAGGCTGAAGCCATC-3' (210-231)
IFNA7	11 / 22 (50%)	5'-GTTCCAGAAGACTCAAGCCATC-3' (250-271)

Reverse PCR primer: R1, 5'-CATAAAACATGATTTAAACCT-TAAAAATAG-3' to detect IFN-α16 mRNA/AS RNA

Gene symbol	Homology	Sequence (Location in the gene)
IFNA16	30 / 30 (100%)	5'-CATAAAACATGATTTAAACCT-TAAAAATAG-3' (839-810)
IFNA4	25 / 30 (83%)	5'-CATAAAAAATAATTTAAATTT-TAAAAATAA-3' (893-864)
IFNA17	23 / 30 (77%)	5'-ATAAAAAAATAATTTAAATCT-TAAAAATAA-3' (878-849)
IFNA1	22 / 30 (73%)	5'-GAACAAAAATAATTTAAATCT-TATGAATAG-3' (811-782)
IFNA2	21 / 31 (68%)	5'-AATAAATAATATTTTAAAAATATTTAAATAG-3' (871-841)
IFNA8	20/ 30 (67%)	5'-GAACAAAACTAATTTAAGCCC-TATAGATAG-3' (870-841)
IFNA21	20 / 30 (67%)	5'-GGACAAAAATAATTTAAATCT-AATAAATAA-3' (878-849)
IFNA10	19 / 30 (63%)	5'-CTCATGATATTACATAAATTT-TAAAAATAA-3' (874-845)'
IFNA14	16 / 30 (53%)	5'-CATGATGCTTCTTTACACTCC-TGAAAACAT-3' (766-737)
IFNA7	15 / 30 (50%)	5'-GATGAACCAGTTTTCAATCCT-TCCTCCTTA-3' (623-594)
IFNA5	14 / 30 (47%)	5'-AGGATCTCATGATTTCTGCTC-TGACAACCT-3' (580-551)
IFNA6	14 / 30 (47%)	5'-AGGATCTCATGATTTCTGCTC-TGACAACCT-3' (523-494)
IFNA13	14 / 30 (47%)	5'-GGGATCTCATGATTTCTGCTC-TGACAACCT-3' (592-563)

IFN-α17 mRNA/AS RNA

Forward PCR primer: F1B, 5'-CTAGGCTGTGATCTGCCTCAGAC-3' to detect IFN-α17 mRNA/AS RNA

Gene symbol	Homology	Sequence (Location in the gene)
IFNA17	23 / 23 (100%)	5'-CTAGGCTGTGATCTGCCTCAGAC-3' (113-135)
IFNA10	23 / 23 (100%)	5'-CTAGGCTGTGATCTGCCTCAGAC-3' (110-132)
IFNA4	22 / 23 (96%)	5'-CTGGGCTGTGATCTGCCTCAGAC-3' (132-154)
IFNA5	22 / 23 (96%)	5'-CTGGGCTGTGATCTGCCTCAGAC-3' (121-143)
IFNA7	22 / 23 (96%)	5'-CTGGGCTGTGATCTGCCTCAGAC-3' (104-126)
IFNA8	22 / 23 (96%)	5'-CTGGGCTGTGATCTGCCTCAGAC-3' (94-116)
IFNA16	22 / 23 (96%)	5'-CTGGGCTGTGATCTGCCTCAGAC-3' (70-92)
IFNA21	22 / 23 (96%)	5'-CTGGGCTGTGATCTGCCTCAGAC-3' (112-134)
IFNA6	21 / 23 (91%)	5'-CTGGACTGTGATCTGCCTCAGAC-3' (64-86)
IFNA2	20 / 23 (87%)	5'-GTGGGCTGTGATCTGCCTCAAAC -3' (132-154)
IFNA1	20 / 23 (87%)	5'-CTGGGCTGTGATCTCCCTGAGAC-3' (131-153)
IFNA13	20 / 23 (87%)	5'-CTGGGCTGTGATCTCCCTGAGAC-3' (133-155)
IFNA14	19 / 23 (83%)	5'-CTGGGCTGTAATCTGTCTCAAAC-3' (108-130)

Reverse PCR primer: R1, 5'-CCATCAAACTCCTCCTGGGGAAG-3' to detect IFN-α17 mRNA/AS RNA

Gene symbol	Homology	Sequence (Location in the gene)
IFNA17	23 / 23 (100%)	5'-CCATCAAACTCCTCCTGGGGAAG-3' (252-230)
IFNA1	22 / 23 (96%)	5'-CCATCAAACTCCTCCTGGGGAAA-3' (270-248)
IFNA6	22 / 23 (96%)	5'-CCATCAAACTCCTCCTGGGGAAA-3' (203-181)
IFNA13	22 / 23 (96%)	5'-CCATCAAACTCCTCCTGGGGAAA-3' (272-250)
IFNA5	21 / 23 (91%)	5'-CCATCAAACTCCTCCTGAGGAAA-3' (260-238)
IFNA10	21 / 23 (91%)	5'-CCATCAAACTCCTCCTGGGGGAT-3' (249-227)
IFNA14	21 / 23 (91%)	5'-CCATCAAATTCCTCCTGGGGAAA-3' (247-225)
IFNA21	21 / 23 (91%)	5'-CCATCAAACTCCTCCTGGGGGAA-3' (251-229)
IFNA8	20 /23 (87%)	5'-TCATCAAACTCCTCCTGGGGGAA-3' (233-211)
IFNA4	20 / 23 (87%)	5'-CCATCAAACTCCTCCTCGGGGAA-3' (271-249)
IFNA16	20 / 23 (87%)	5'-CCATCAAACACCTCCTGGGGGAA-3' (209-187)
IFNA7	19 / 23 (83%)	5'-CCATCAAACTCCTCCTCTGGGAA-3' (243-221)
IFNA2	18 / 23 (78%)	5'-TTGCCAAACTCCTCCTGGGGAAA-3' (271-249)

IFN-α21 mRNA/AS RNA

Forward PCR primer: F1B, 5'-TTTTGTCCATGTAATATTATGTGT-3' to detect IFN-α21 mRNA/AS RNA

Gene symbol	Homology	Sequence (Location in the gene)
IFNA21	24 / 24 (100%)	5'-TTTTGTCCATGTAATATTATGTGT-3' (871-894)
IFNA8	20 / 24 (83%)	5'-TTTTGTTCATATTATATTATGTGA-3' (863-886)
IFNA17	19 / 24 (79%)	5'-ATTTTTTTATGTAATATCATGTGT-3' (869-892)
IFNA1	18 / 24 (75%)	5'-TTTTGTTCATATAACGTCATGTGC-3' (804-827)
IFNA2	18 / 24 (75%)	5'-TTTTGTTCATATTATGTCATGTGC-3' (895-918)
IFNA4	17 / 24 (71%)	5'-TATTTTTTATGTGATATCATGAGT-3' (883-906)
IFNA10	17 / 24 (71%)	5'-TGTGGTTAATGTAACAATATATGT-3' (886-909)
IFNA16	17 / 24 (71%)	5'-CATGTTTTATGTAATATCATGTGT-3' (829-852)
IFNA13	15 / 24 (63%)	5'-TCTTTACCACAAAAGATTCATCTGC-3' (338-362)
IFNA5	12 / 24 (50%)	5'-TGTCCTCCATGAGATAATCCAGCA-3' (291-314)
IFNA7	12 / 24 (50%)	5'-AATTTTCCACTGAACTTTACCAGC-3' (360-383)
IFNA14	11 / 24 (46%)	5'-TGTCCTCCATGAGATGATGCAGCA-3' (278-301)
IFNA6	10 / 24 (42%)	5'-TGTCCTCCATGAGGTGATTCAGCA-3' (234-257)

Reverse PCR primer: R1, 5'-TAAAGAATAAACAAAT-AATATAAGAAGTTT-3' to detect IFN-α21 mRNA/AS RNA

Gene symbol	Homology	Sequence (Location in the gene)
IFNA21	30 / 30 (100%)	5'-TAAAGAATAAACAAAT-AATATAAGAAGTTT-3' (1005-976)
IFNA4	20 / 30 (67%)	5'-TTAAAAATAAATAAAT-ATTTAAATAAATAG-3' (873-844)
IFNA8	20 / 30 (67%)	5'-TAAAGAATAAATAAAG-AATTTTTCTATAGT-3' (990-961)
IFNA2	19 / 30 (63%)	5'-TAAAGAATAAACAAGT-ACAAAAGTTTCCAT-3' (1030-1001)
IFNA17	19 / 31 (61%)	5'-TAATGAAAAAGGAAATTAATATATTGGCTAA-3' (966-936)
IFNA1	18/ 30 (60%)	5'-AAATAAATATTTAAAT-AGATAAATCAGTTT-3' (779-750)
IFNA10	17/ 30 (57%)	5'-AAATAATTAAATAAAT-ATTTAAACAAAAGA-3' (850-821)
IFNA16	17 / 30 (57%)	5'-AGTTAAATAAATAAAT-ATTTAAATAAATAG-3' (811-782)
IFNA13	16 / 30 (53%)	5'-ATATGAGTCAATAAGA-ATTGTTTCATGTTG-3' (678-649)
IFNA7	15 / 30 (50%)	5'-TGTTGAAAAAGAGAAG-GATCTCATGATTTC-3' (577-548)
IFNA14	15/ 30 (50%)	5'-TTTACACTCCTGAAAA-CATTTGAAAATTTT-3' (755-726)
IFNA5	14 / 30 (47%)	5'-TGCTGATAAAGAGAAG-GATCTCATGATTTC-3' (594-565)
IFNA6	12 / 30 (40%)	5'-TCCTTCCTCCTTAACC-TTTCTTGCAAGTTT-3' (566-537)
