# Supplementary material for: Interferon-alpha competing endogenous RNA network antagonizes microRNA-1270
Source: Cell Mol Life Sci. 2015 Mar 7;72(14):2749–61. doi: 10.1007/s00018-015-1875-5 (PMC4477080; doi:10.1007/s00018-015-1875-5)
Supplement: Supplementary file 3 — Supplementary material 3 (PDF 69 kb) [file 18_2015_1875_MOESM3_ESM.pdf]

Supplementary Table 4 Base paring of miR-1270 with the putative MRE-1270s on IFN-α1 AS/exons 0.1 and 1.1.

```
Position: -3101 - -3077 (exon 0.1)                                                      ΔG: -18.8 kcal/mol
               5'    UU  GGCA              G 3'
                   CAC  GU    UUCUG AUCUUUAG
                   GUG  CG    AAGGU UAGAGGUC
MiR-1270       3' U   U   AG       A          5'
```

```
Position: -121 - -101 (exon 1.1/DSR)                                                    ΔG: -16.5 kcal/mol
               5' A     C      AU        A 3'
                   AUAUA C CUUU    UCUCUA
                   UGUGU G GAAG    AGAGGU
MiR-1270       3'        C A    GUAU      C 5'
```

```
Position:  556 - 507  (exon 1.1/stop-SL2 region)                                        ΔG: -21.2 kcal/mol
               5' G    AAG   UGUAUU      CUGUCAGAUAGAGA   AU       A 3'
                   GCAC   GGC       UCUUCU              GUG  UCUUCGG
MiR-1270       3'  UGUG   UCG       AGAAGG              UAU  AGAGGUC  5'
```

```
Position:  436 - 412  (exon 1.1/SL2R)                                                   ΔG: -28.8 kcal/mol
5'             5' C      G       A   A     C 3'
                   ACACAG  CUUCCA GUC UUCAG
                   UGUGUC  GAAGGU UAG AGGUC
MiR-1270       3'       GA       A           5'
```

```
Position:  186 - 160  (exon 1.1/SL1-ATG region)                                         ΔG: -23.6 kcal/mol
5'              5'A    CA  G  C        A     G 3'
                   GCAU  AG UC UCC UGUU UCCAG
                   UGUG  UC AG AGG AUAG AGGUC
MiR-1270        3'         G  A   U            5'
```
