# Supplementary figures and images for: Interferon-alpha competing endogenous RNA network antagonizes microRNA-1270
Source: Cell Mol Life Sci. 2015 Mar 7;72(14):2749–61. doi: 10.1007/s00018-015-1875-5 (PMC4477080; doi:10.1007/s00018-015-1875-5)

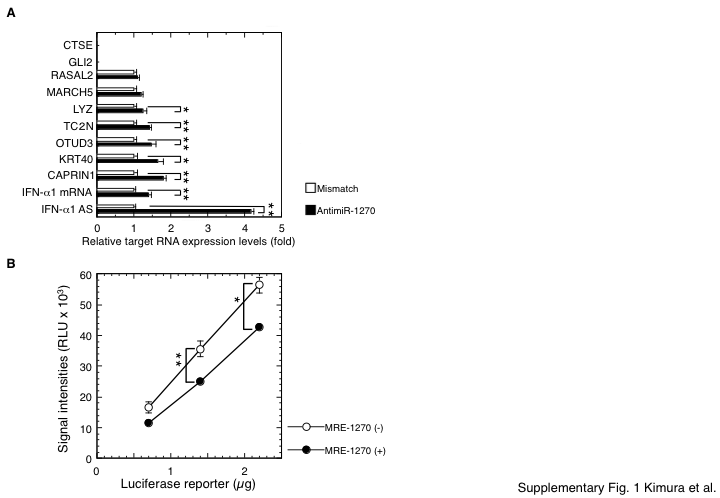

Supplement: Supplementary file 4 — Supplementary material 4 (TIFF 1402 kb) [file 18_2015_1875_MOESM4_ESM.tif]

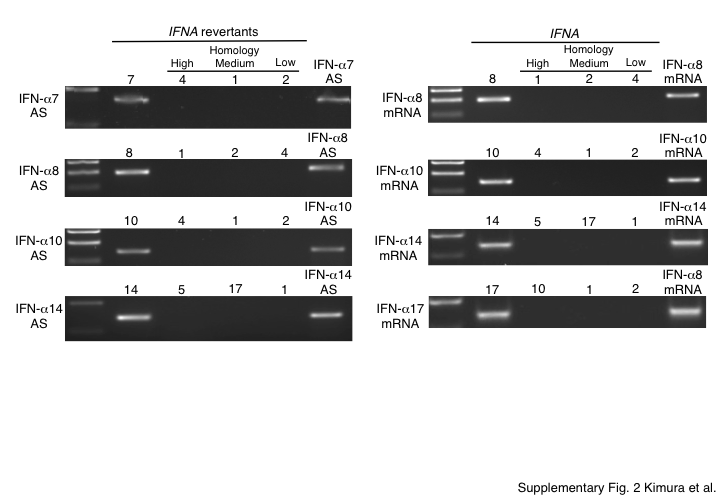

Supplement: Supplementary file 5 — Supplementary material 5 (TIFF 1402 kb) [file 18_2015_1875_MOESM5_ESM.tif]
