# Supplementary material for: Interferon-alpha competing endogenous RNA network antagonizes microRNA-1270
Source: Cell Mol Life Sci. 2015 Mar 7;72(14):2749–61. doi: 10.1007/s00018-015-1875-5 (PMC4477080; doi:10.1007/s00018-015-1875-5)
Supplement: Supplementary file 6 — Supplementary material 6 (PDF 93 kb) [file 18_2015_1875_MOESM6_ESM.pdf]

Supplementary Table 1 List of primers used in this study.

MRE-1270 mapping assay[a]

For construction of pEF-*Luc*-IFN-α1-SL2/ΔBSL

ΔBSL-invF1, 5'-agtTCATCTGCTGCTTGGGATGAG-3' (human chromosomal *IFNA1* coding segment nt 353-373)

ΔBSL-invR1, 5'-agtTGGATCAGCTCATGaagcttctag-3' (human chromosomal *IFNA1* coding segment nt 321-308)

For construction of pEF-*Luc*-IFN-α1-AS/ exon0.1

0.1F, 5'-gctctagaCACTTGTGGCATTCTGATCTTT-3' (human chromosome 9 nt 21444417-21444395)

0.1R, 5'-gctctagaGCACATTCTCTCTCTGGCTTTA-3' (human chromosome 9 nt 221444300-21444322)

For construction of pEF-*Luc*-IFN-α1-3' UTRR-DSR

3' UTR-F: 5'-cccaagcttCATCTGGTCCAA CATGAAAACAATTC-3' (human chromosomal *IFNA1* coding segment nt 638-663)

DS-R: 5'-gctctagaGCAAGAGAGTAGGAAGTAG ATTTATTGTAT-3' (nt 188-159 from the polyadenylation site of *IFNA1*)

For construction of pEF-*Luc*-IFN-α1-(SL2-stop codon)R

SL2-stop F: 5'-gctctagaTGATGCAGGAGGAGAGGGT-3' (human chromosomal *IFNA1* coding segment nt 435-453)

SL2-stop R: 5'-gctctagaTTATTCCTTCCTCCTTAATCTTTCTT-3' (human chromosomal *IFNA1* coding segment nt 637-612)

For construction of pEF-*Luc*-IFN-α1-(ATG-SL1)R

ATG-SL1 F: 5'- gctctagaATGGCCTCGCCCTTTGCTTTAC -3' (human chromosomal *IFNA1* coding segment nt 68-89)

ATG-SL1 R: 5'-gctctagaGAGGACAGAGATGGCTGGAGCC-3' (human chromosomal *IFNA1* coding segment nt 307-286)

For construction of pEF-*Luc*-CAPRIN1 3'-UTR

CAPRIN1 F: 5'-gctctagaTGGTGTTCAACAGCTAGCAG-3' (Homo sapiens *CAPRIN1*, transcript variant 1, mRNA nt: 3474-3493)

CAPRIN1 R: 5'-gctctagaAAATGCCTCCAGGAGAAACG-3' (Homo sapiens *CAPRIN1*, transcript variant 1, mRNA nt: 3632-3613)

IFN-α superfamily cross reactivity assay[b]

For construction of phuIFN-α7 expression vector[a]

α7F, 5'-cccaagcttTACCCACCTCAGGTAGC-3' (*IFNA7* nt 1-17)

α7R, 5'-tgctctagaGAAAACATTTGGAAATTTTGATTCAACTTG-3' (*IFNA7* nt 737-708)

For construction of phuIFN-α8 expression vector

α8F, 5'-cccaagcttACCAGCTCAGCAGCATCCA-3' (*IFNA8* nt 1-19)

α8R, 5'-cccaagcttACCATCCATTCTTTAATCAGTTTGCA-3' (*IFNA8* nt 1039-1014)

For construction of phuIFN-α10 expression vector

α10flankF, 5'-AGAAAACCTAGAGGCCGAAGTTCAAGGT-3' (nt -22-6 from the transcription initiation site of *IFNA10*)

α10F, 5'-cccaagcttCAAGGTTATCCATCTCAAGTA-3' (*IFNA10* nt 1-21)

α10R, 5'-gctctagaGTATAGTAAAAATTTAATGAAAAAGGAAATTA-3' (*IFNA10* nt 963-932)

For construction of phuIFN-α14 expression vector

α14F, 5'-cccaagcttGTTACCCCTCATCAACCAGCC-3' (*IFNA14* nt 1-21)

α14R, 5'-tgctctagaTGCACAGGTATACATGATGCTTCTTTACAC-3' (*IFNA14* nt 778-749)

Strand-specific RT-PCR

For detection of IFN-α2 mRNA/AS RNA

RT primer: R2, 5'-GATCTCATGATTTCTGCTCTGAC-3' *(IFNA2* nt 586-564) and F1, 5'-TGGTGCTCAGCTGCAAGTCAAGC-3' (*IFNA2* nt 103-125), respectively.

PCR primer pair: F1B, 5'-GTGGGCTGTGATCTGCCTCAAAC-3' (*IFNA2* nt 132-154)/R1, 5'-AGGGATGGTTTCAGCCTTTTG-3' (*IFNA2* nt 299-279)

For detection of IFN-α4 mRNA/AS RNA

RT primer: R2, 5'-GATCTCATGATTTCTGCTCTGAC-3' *(IFNA4* nt 589-567) and F1B, 5'-ACATGATTTCGGATTCCCCGAGGAGGAG-3' (*IFNA4* nt 236-263), respectively.

PCR primer pair: *INFA4* F1B as above /R1, 5'-TTTTTCTAGGAGGCTCTGTTCCCAAGCAGC-3' (*IFNA4* nt 389-360)

For detection of IFN-α5 mRNA/AS RNA

RT primer: R2, 5'-GATCTCATGATTTCTGCTCTGAC-3' (*IFNA5* nt 578-556) and F1, 5'-TGGTGGTGCTCAACTGCAAGTCAAT-3' (*IFNA5* nt 89-113), respectively.

PCR primer pair: F1B, 5'-CAGACCCACAGCCTGAGTAAC-3' (*IFNA5* nt 139-159) /R1, 5'-GAATTTGTCTAGAAGTGTCTCATCCCAAGT-3' (*IFNA5* nt 381-352)

For detection of IFN-α6 mRNA/AS RNA

RT primer: R2, 5'-GATCTCATGATTTCTGCTCTGAC-3' (*IFNA6* nt 521-499) and F1, 5'-TGGTGCTCAGCTGCAAGTCAAGC-3' (*IFNA6* nt 35-57), respectively.

PCR primer pair: F1B, 5'-CTGGGTCACAGGAGGACCATGAT-3' (*IFNA6* nt 94-116) /R1, 5'-CTGAATCACCTCATGGAGGACAGAG-3' (*IFNA6* nt 255-231)

For detection of IFN-α7 mRNA/AS RNA

RT primer: R2, 5'-GATCTCATGATTTCTGCTCTGAC-3' (*IFNA7* nt 561-539) and F1, 5'-5'-TACCCACCTCAGGTAGCCTAGTGAT -3' (*IFNA7* nt 1-25), respectively.

PCR primer pair: F2, 5'-GTACTCAGCTACAAATCCATCTGCTCTC-3' (*IFNA7* nt 77-104) /R1, 5'-CCTCTGGGAATCTGAATTCATGTCTGTCC-3' (*IFNA7* nt 230-202)

For detection of IFN-α8 mRNA/AS RNA

RT primer: R2, 5'-ACCATCCATTCTTTAATCAGTTTG-3' (*IFNA*8 nt 1039-1016) and commonF, 5'-CTCTGGGCTGTGATCTGCCTCAGAC-3' (*IFNA8* nt 92-116), respectively.

PCR primer pair: F1B, 5'-CTATCTATAGGGCTTAAATTAGTTTTGTTC-3' (*IFNA8* nt 841-870) /R1, 5'-CATTCTTTAATCAGTTTGCACAATC-3' (*IFNA8* nt 1033-1009)

For detection of IFN-α10 mRNA/AS RNA

RT primer: commonR, 5'-GTATAGTAAAAATTTAATGAAAA-3' (*IFNA10* nt 963-941) and commonF, 5'-CTCTGGGCTGTGATCTGCCTCAGAC-3' (*IFNA10* nt 108-132)

PCR primer pair: F1B, 5'-TCACTTCTATAACCACGACGC-3' (*IFNA*10 nt 696-716) /R1, 5'-CGACTCATGATATTACATAAATTTTAAA-3' (*IFNA*10 nt 877-850)

For detection of IFN-α14 mRNA/AS RNA

RT primer: R2, 5'-GATCTCATGATTTCTGCTCTGAC-3' (*IFNA14* nt 565-543) and F1, 5'-TGGTGCTCAGCTGCAAGTCAAGC-3' (*IFNA14* nt 79-101), respectively.

PCR primer pair: F1B, 5'-CTGGGCTGTAATCTGTCTCAA-3' (*IFNA14* nt 108-128) /R1, 5'-AGAGATGGCTTGAGCTTTCTG-3' *(IFNA14* nt 278-258)

For detection of IFN-α16 mRNA/AS RNA

RT primer: commonR, 5'-GTATAGTAAAAATTTAATGAAAA-3'' (*IFNA16* nt 939-917) and commonF, 5'-CTCTGGGCTGTGATCTGCCTCAGAC-3' (*IFNA16* nt68- 92), respectively.

PCR primer pair: F1B, 5'-TTTTCAGGAGTGTAAAGAAGCA-3' (*IFNA16* nt 699-720) /R1, 5'-CATAAAACATGATTTAAACCTTAAAAATAG-3' (*IFNA16* nt 839-810)

For detection of IFN-α17 mRNA/AS RNA

RT primer: R2, 5'-GATCTCATGATTTCTGCTCTGAC-3'' (*IFNA17* nt 560-548) and F1B, 5'-CTAGGCTGTGATCTGCCTCAGAC-3' *(IFNA17* nt 113-135), respectively.

PCR primer pair: F1B as above (*IFNA17* nt 113-135) /R1, 5'-CCATCAAACTCCTCCTGGGGAAG-3' (*IFNA17* nt 252-230)

For detection of IFN-α21 mRNA/AS RNA

RT primer: R1, 5'-TAAAGAATAAACAAATAATATAAGAAGTTT-3' *(IFNA21* nt 1005-976) /commonF, 5'-CTCTGGGCTGTGATCTGCCTCAGAC-3' (*IFNA21* nt 110-134), respectively.

PCR primer pair: F1B, 5'-TTTTGTCCATGTAATATTATGTGT-3' (*IFNA21* nt871-894) /R1, as above (*IFNA21* nt 1005-976)

For detection of CAPRIN1 mRNA

RT primer: R1, 5'-ATAGCTGAGCACCTACTGGAAG-3' (*CAPRIN1* nt 3928-3907)

PCR primer pair: F1, 5'-TGGTGTTCAACAGCTAGCAG-3' (*CAPRIN1* nt: 3474-3493) /R2, 5'-AAATGCCTCCAGGAGAAACG-3' (*CAPRIN1* nt: 3632-3613)

For detection of CTSE mRNA

RT primer: R1, 5'-AGAGAGGCACCAGCATTATCTG-3' (*CTSE* nt: 2153-2132)

PCR primer pair: F1, 5'-TTATCTACACTGCTGCCCACTC-3' *(CTSE* nt: 1707-1728) /R2, 5'-TGGGACAATGGGAGATGCTTG-3' (*CTSE* nt: 1858-1838)

For detection of GLI2 mRNA

RT primer: R1, 5'-AAGGGGGAAGCAGATAGACAAG-3' (*GLI2* nt: 6642-6621)

PCR primer pair: F1, 5'-TGAAAGCCAGGGAACATTGC-3' (*GLI2* nt: 6357-6376) /R2, 5'-AATTTGGCTGGACGACTCAC-3' (GLI2 nt: 6508-6489)

For detection of KRT40 mRNA

RT primer: R1, 5'-CCACGCTAAAGGAATAAAACACG-3' (*KRT40* nt: 1707-1685)

PCR primer pair: F1, 5'-AGCAGGTAGAGGAATCAAAGCC-3' (*KRT40* nt: 1469-1490) /R2, 5'-AGAATGCTTTATGGGCTTCCAG-3' (*KRT40* nt: 1665-1644)

For detection of LYZ mRNA

RT primer: R1, 5'-GAGATGACTGAACAGATGAAGGC-3' (*LYZ* nt: 1231-1209)

PCR primer pair: F1, 5'-GAAGCAGGAGCAAAATATGGC-3' (*LYZ* nt: 616-636) /R2, 5'-AAAAAGAACGGAATGTACTGGAG-3' (*LYZ* nt: 785-763)

For detection of MARCH5 mRNA

RT primer: R1, 5'-GCAAAGTTAGCAAGGCAGTG-3' (*MARCH5* nt: 3840-3821)

PCR primer pair: F1, 5'-GGGGACAAATGAAAGAGATGATG-3' (*MARCH5* nt: 3562-3584) /R2, 5'-TTTTCAGCCACTTTGGAGATAAG-3' (*MARCH5* nt: 3749-3727)

For detection of OTUD3 mRNA

RT primer: R1, 5'-AACTAACAGCACCTGCATCC-3' (*OTUD3* nt: 1968-1949)

PCR primer pair: F1, 5'-ATCAGACTTCAGTGACCCTAGG-3' (*OTUD3* nt: 1628-1649) /R2, 5'-AGCATATTCCTGAGTTGGAGATC-3' (*OTUD3* nt: 1809-1787)

For detection of RASAL2 mRNA

RT primer: R1, 5'-GGCCAGAGTGGGGTTTTTAAAC-3' (*RASAL2* nt: 5117-5096)

PCR primer pair: F1, 5'-ACCCCACCAAGCTTTCCATC-3' (*RASAL2* nt: 4552-4571) /R2, 5'-ACCACATCGCCATTGTGAAG-3' (*RASAL2* nt: 4722-4703)

For detection of TC2N mRNA

RT primer: R1, 5'-AGATGGCACCTAGGGAAAAGAG-3' (*TC2N* nt: 3183-3162)

PCR primer pair: F1, 5'-GGAGAAAGTTTGTGCCCTGTG-3' (*TC2N* nt: 2392-2412) /R2, 5'-TTGAGGGAAGAAAACAGCAAAG-3' (*TC2N* nt: 2585-2564)

AntimiR oligonucleotides[c]

antimiR-1270: 5'-T(L)^A(L)^T(L)^5(L)^T(L)^5(L)^5(L)^A(L)-3'

LNA mismatch: 5'-T(L)^G(L)^G(L)^A(L)^G(L)^A(L)^T(L)^A(L)-3'

[a]The flanking restriction sites are in lower case type

[b]The flanking restriction sites are in lower case type.

[c](L) and (5) indicate LNA nucleoties and LNA methylcytosine, respectively. ^ shows a phosphorothioate bond.
